# Erythrocyte adenosine A2B receptor prevents cognitive and auditory dysfunction by promoting hypoxic and metabolic reprogramming

**DOI:** 10.1371/journal.pbio.3001239

**Published:** 2021-06-17

**Authors:** Qingfen Qiang, Jeanne M. Manalo, Hong Sun, Yujin Zhang, Anren Song, Alexander Q. Wen, Y. Edward Wen, Changhan Chen, Hong Liu, Ying Cui, Travis Nemkov, Julie A. Reisz, George Edwards III, Fred A. Perreira, Rodney E. Kellems, Claudio Soto, Angelo D’Alessandro, Yang Xia

**Affiliations:** 1 Department of Otolaryngology Head and Neck Surgery, Xiangya Hospital, Central South University, Changsha, Hunan, China; 2 Department of Biochemistry and Molecular Biology, The University of Texas McGovern Medical School, Houston, Texas, United States of America; 3 University of Texas MD Anderson UTHealth Graduate School of Biomedical Sciences, Houston, Texas, United States of America; 4 University of California at San Diego, La Jolla, California, United States of America; 5 University of Texas Southwestern Medical School, Dallas, Texas, United States of America; 6 Department of Dermatology, Xiangya Hospital, Central South University, Changsha, Hunan, China; 7 Department of Biochemistry and Molecular Genetics, University of Colorado School of Medicine, Aurora, Colorado, United States of America; 8 Department of Neurology, The University of Texas McGovern Medical School, Houston, Texas, United States of America; 9 Huffington Center on Aging, Baylor College of Medicine, Houston, Texas, United States of America; Buck Institute for Research on Aging, UNITED STATES

## Abstract

Hypoxia drives aging and promotes age-related cognition and hearing functional decline. Despite the role of erythrocytes in oxygen (O_2_) transport, their role in the onset of aging and age-related cognitive decline and hearing loss (HL) remains undetermined. Recent studies revealed that signaling through the erythrocyte adenosine A2B receptor (ADORA2B) promotes O_2_ release to counteract hypoxia at high altitude. However, nothing is known about a role for erythrocyte ADORA2B in age-related functional decline. Here, we report that loss of murine erythrocyte–specific ADORA2B (e*Adora2b*^−/−^) accelerates early onset of age-related impairments in spatial learning, memory, and hearing ability. *eAdora2b^-/-^* mice display the early aging-like cellular and molecular features including the proliferation and activation of microglia and macrophages, elevation of pro-inflammatory cytokines, and attenuation of hypoxia-induced glycolytic gene expression to counteract hypoxia in the hippocampus (HIP), cortex, or cochlea. Hypoxia sufficiently accelerates early onset of cognitive and cochlear functional decline and inflammatory response in e*Adora2b*^−/−^ mice. Mechanistically, erythrocyte ADORA2B-mediated activation of AMP-activated protein kinase (AMPK) and bisphosphoglycerate mutase (BPGM) promotes hypoxic and metabolic reprogramming to enhance production of 2,3-bisphosphoglycerate (2,3-BPG), an erythrocyte-specific metabolite triggering O_2_ delivery. Significantly, this finding led us to further discover that murine erythroblast ADORA2B and BPGM mRNA levels and erythrocyte BPGM activity are reduced during normal aging. Overall, we determined that erythrocyte ADORA2B–BPGM axis is a key component for anti-aging and anti-age–related functional decline.

## Abstract 2

Hypoxia drives aging and promotes age-related functional decline in cognition and hearing. This study shows that signaling through the erythrocyte adenosine A2B receptor promotes metabolic reprogramming, leading to increased production of 2,3-bisphosphoglycerate and lowering hypoxia-induced inflammatory responses in the hippocampus, cortex and cochlea during aging.

## Introduction

With an increasing trend in longevity worldwide over the past 2 centuries, the mean age of the population is rising rapidly. Age-related functional decline profoundly affects elderly’s ability to perform daily living activities independently and are highly related to age-related diseases (ARDs), resulting in a heavy burden on modern society with rising healthcare expenditures [1,2]. Age-related functional decline such as decline of cognitive and hearing function (hearing loss [HL]) is the most common, least understood, and poorly treated age-related functional decline [3,4]. At present, substantial research in age-related decline has largely focused on genetics and gene expression changes in end organs. However, gene expression changes in end organs do not fully explain how cognition and hearing decline develop during aging. Thus, here, we sought to define the cellular, molecular, and metabolic basis underlying age-related decline with a goal to develop novel approaches to define aging and early onset of age-related decline and ameliorate the progression of age-related decline.

Recently, considerable evidence has supported the notion of cognition decline and HL as systemic metabolic diseases [5,6]. In particular, the brain is majorly dependent on glucose oxidization to generate sufficient ATP to maintain neuronal function and survival [7,8]. It is well known that oxygen (O_2_) deprivation for as short as a few minutes can trigger significant brain dysfunction and that chronic hypoxia can lead to irreversible brain injury and permanent impairment of cognition. This underpins the concept that hypoxia is an early driver of cerebrometabolic abnormalities, onset of age-related cognitive decline and HL, and their progression. It has long been speculated that aging is accompanied with a general decrease in O_2_ supply to tissues [9] and leads to a series of responses such as activation of macrophages to release more pro-inflammatory chemokines and cytokines [10], thereby promoting immune cell activation [11] and increased inflammation [12], which could result in decline in cognition and hearing. Altogether, this whole process is often referred to as “inflammaging” [13].

Notably, adenosine signaling is reported to be involved in the regulation of brain function and decline during the aging process. Extracellular adenosine orchestrates a physiological and pathological hypoxic response by the activation of 4 specific cell surface adenosine receptors. The levels of extracellular adenosine in the brain increase with age [14,15], which could activate adenosine receptors in the brain resulting in behavioral consequences. Among 4 adenosine receptors, adenosine A1 receptor (ADORA1) and adenosine A2A receptor (ADORA2A) are both highly expressed and distributed widely in neurons and gliocytes throughout the brain [16,17]. ADORA1 and ADORA2A have been shown to have both a presynaptic and postsynaptic neuromodulation effect [18]. Meanwhile, regulation of synaptic plasticity by ADORA1 and ADORA2A has also been reported [19]. It is well accepted that ADORA1 functions as neuroprotective, whereas ADORA2A functions as neurodegenerative [20–22]. On the other hand, adenosine A3 receptor (ADORA3) and adenosine A2B receptor (ADORA2B) have widespread distribution in the brain but are restricted to a low level of expression [16]. Most studies about adenosine receptors in cognitive decline have focused on neurons or immune cells of the brain. Although the erythrocyte is the only cell type carrying and delivering O_2_ to every single cell type within our body, the functional role of erythrocytes in age-related cognitive decline and HL is unrecognized.

Recent studies demonstrated that red blood cell (RBC) metabolic adaptation to high-altitude hypoxia results in increased O_2_ delivery to counteract tissue hypoxia [23]. The results showed that this process is regulated by elevated adenosine signaling via ADORA2B, which, in turn, induced the activation of AMP-activated protein kinase (AMPK). AMPK activation phosphorylates and activates bisphosphoglycerate mutase (BPGM), the rate-limiting enzyme of the Rapoport–Luebering shunt, thus fueling the synthesis of 2,3-bisphosphoglycerate (2,3-BPG). Most importantly, 2,3-BPG is an allosteric modulator of hemoglobin (HGB), which favors O_2_ release to counteract hypoxia [24]. In contrast to the high altitude–induced erythrocyte hypoxic adaptive response, erythrocyte 2,3-BPG and ATP levels decline with age and are further reduced in age-matched individuals with Alzheimer disease (AD) [25–27]. However, the function and underlying mechanisms responsible for decreased erythrocyte ATP and 2,3-BPG in aging and progression of age-related functional decline remain undetermined, and approaches for the restoration of erythrocyte ATP and 2,3-BPG levels in aging have not yet been developed. In light of the above, here, we conducted mouse genetic studies to determine the role of erythrocyte ADORA2B in early onset of age-related decline including cognition, memory, and cochlear functional decline. As a result, we identified a series of underlying mechanisms that highlight therapeutic opportunities to increase tissue oxygenation and slow functional decline progression by targeting the erythrocyte ADORA2B-BPGM signaling axis.

## Results

### Genetic ablation of erythrocyte ADORA2B leads to early cognitive and cochlear functional decline

**Fig 1:**
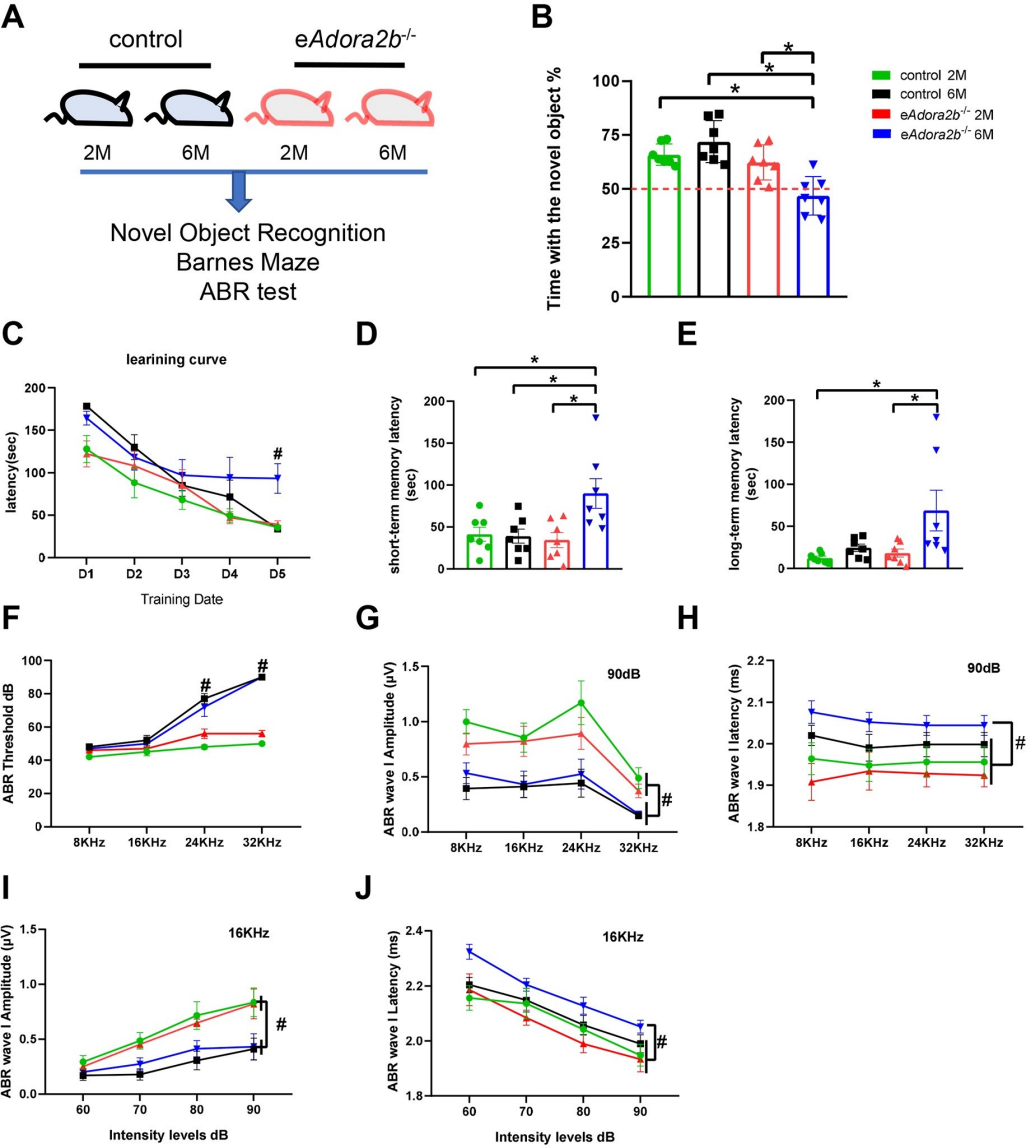
Erythrocyte ADORA2B deficiency impaired cognitive and cochlear function at 6 months. **(A)** Schematic representation of experimental design. Two-month-old and 6-month-old control and e*Adora2b*^−/−^ mice cognitive and cochlear functions were evaluated by NOR, BM, and ABR test. **(B)** Percentage of time exploring the novel object was quantified in final trial of NOR, and a value lower than 50% demonstrated impaired cognitive abilities. Data are shown as mean ± SEM. *n* = 7 mice/group. *P < 0.05. **(C)** Learning curve was measured in the 4 groups for 5 consecutive days as the primary latency to find escape box in seconds. Data are expressed as mean ± SEM. *n* = 7 mice/group. #*P* < 0.05 compared to other 3 groups. **(D, E)** Short-term memory (D) and long-term memory (E) were measured as the primary latency in seconds for mice to find the escape box. Data are shown as mean ± SEM. *n* = 7 mice/group. *P < 0.05. **(F–J)** ABR test was applied to measure HL of the 4 groups. Threshold (F) was defined as the lowest intensity to detect Waves I–V. Data are shown as mean ± SEM. *n* = 5 mice/group. #*P* < 0.05, e*Adora2b*^−/−^ 6M and control 6M vs. e*Adora2b*^−/−^ 2M and control 2M. Average Wave I amplitude (G) and latency (H) were measured in response to 90 dB. Data are shown as mean ± SEM. *n* = 5 mice/group. #*P* < 0.05. Average Wave I amplitude (I) and latency (J) were measured in response to 16 KHz. Data are shown as mean ± SEM. *n* = 5 mice/group. #*P* < 0.05. (G) and (I) were assessed by e*Adora2b*^−/−^ 6M and control 6M vs. e*Adora2b*^−/−^ 2M and control 2M. (H) and (J) were assessed by e*Adora2b*^−/−^ 6M compared to other 3 groups. *P were measured by 1-way ANOVA with Tukey multiple comparison test. #*P* were tested by 2-way ANOVA with Tukey post hoc test. For all graphs, numerical data underlying plots are provided in S1 Data. ABR, auditory brainstem response; BM, Barnes Maze test; ADORA2B, adenosine A2B receptor; HL, hearing loss; NOR, novel object recognition.

To determine if erythrocyte ADORA2B affects cognitive and cochlear function during aging (2 months versus 6 months), we applied multiple assays to evaluate learning and memory (novel object recognition [NOR] and the Barnes Maze test [BM]) and hearing (auditory brainstem response [ABR]) in controls and *Adora2b*^f/f^/EpoR-Cre^+^ mice (e*Adora2b*^−/−^) with erythrocyte-specific ablation of the Adora2b gene (Fig 1A). Mice were all bred under normoxia. First, mice lacking erythrocyte ADORA2B showed no obvious deficits in survival or fertility compared to *Adora2b^f/f^* littermate controls. Moreover, genetic ablation of ADORA2B in erythrocytes had no effects on *Adora2b* mRNA levels in either brain or cochlea of e*Adora2b*^−/−^ mice (S1A Fig). Additionally, there was no significant difference in body weights (BWs), spleen weight over body weight ratio (SPL/BW), total RBC number, HGB, reticulocytes, mean corpuscular hemoglobin (MCH), mean corpuscular volume (MCV), white blood cell (WBC), and monocytes between controls and e*Adora2b*^−/−^ mice from 2 months old up to 6 months old. Thus, these findings rule out the possibility that a decline of memory and hearing in e*Adora2b*^−/−^ mice at 6 months old is due to insufficient erythropoiesis (S1C–S1L Fig).

NOR is a common test to define nonspatial learning and memory. Using this assay, we did not see a significant difference in NOR between controls and *eAdora2b*^−/−^ mice at 2 months of age. However, at 6 months of age, e*Adora2b*^−/−^ mice showed less interest in investigating the novel object compared to age-matched controls (Fig 1B). These results indicate that ablation of erythrocyte ADORA2B induces early age-associated impairments in cognitive abilities by 6 months of age.

We next assessed spatial learning and memory using the BM. Similar to NOR analysis, e*Adora2b*^−/−^ and control mice did not show differences at 2 months of age. However, at 6 months of age, the e*Adora2b*^−/−^ mice took more time to find the escape box from day 3 to day 5 during the training period comparing to age-matched controls, especially on day 5 (Fig 1C). The 6-month-old e*Adora2b*^−/−^ mice performed worse than controls and e*Adora2b*^−/−^ at 2 months when evaluated for short-term memory (Fig 1D) and long-term memory (Fig 1E). There were no obvious differences of NOR and the BM between males and females of e*Adora2b*^−/−^ and control mice at 2 months and 6 months of age, respectively (S1C and S1D Fig). Overall, these results indicate that erythrocyte ADORA2B plays an important role in cognitive function including spatial and nonspatial learning and memory abilities.

Next, we assessed tone-burst stimuli ABR. The ABR test evaluates the sum of neuronal activity in response to sound stimulation. Earlier studies [28,29] showed that wild-type (WT) mice on the C57/Bl6 genetic background displayed HL by 6 months of age. Similarly, 6-month-old e*Adora2b*^−/−^ and control mice exhibited high-frequency HL in the threshold test compared to the 2-month-old mice, especially at 24 KHz and 32 KHz (Fig 1F). However, there was no significant difference between control and e*Adora2b*^−/−^ mice at either age.

ABR waves represent the summated electrical transmission response upon stimulation, starting from the spiral ganglion neurons (SGNs) and auditory nerve (AN) (Wave I) to the ascending auditory pathway (Waves II to V). ABR also represent the speed of transmission (latency) between these auditory structures, which is measured by the time difference between the onset of peaks from each wave. Thus, to determine if eADORA2B oblation impaired firing of SGN and electrical neuronal transduction of SGN and AN, we chose 90-dB intensity and 16 KHz, which arouse all groups’ responses to sound stimuli, to accurately measure amplitude and latency of Wave I, respectively. Consistent with the threshold results, both 6-month-old groups (control and e*Adora2b*^−/−^ mice) demonstrated diminished Wave l amplitude in response to a range of sound frequencies and intensities (Fig 1G and 1I), although no significant differences were observed between control and e*Adora2b*^−/−^ mice. However, at 6 months of age, the e*Adora2b*^−/−^ mice showed increased latency of Wave l compared to the other 3 groups in response to both different frequencies and intensities (Fig 1H and 1J), indicating an impairment of electrical neuronal transduction of SGN and AN. Similar to behavior test results, there were no obvious differences in the ABR threshold between males and females of e*Adora2b*^−/−^ and control mice at 2 months or 6 months of age (S1E Fig). Thus, erythrocyte ablation of ADORA2B results in an early cochlear functional decline characterized by decreased transduction speed of SGN and AN. Altogether, we concluded that genetic ablation of erythrocyte ADORA2B accelerates cognitive and cochlear functional decline at a relatively young age.

### Genetic ablation of erythrocyte ADORA2B induces activation of immune cells and inflammatory response and impaired hypoxic adaption in hippocampus, cortex, and cochlea

Cognitive decline is frequently associated with the impairment of neuronal and immune cells in the cerebral cortex (CTX) and hippocampus (HIP) [30,31]. Thus, we chose to focus on neurons, astrocytes, and microglia in these regions by performing immunofluorescence (IF) staining and microscopic analysis and quantification. First, IF staining of neuron-specific antigen NeuN (S2A and S2B Fig) and the astrocyte antigen GFAP (S2C Fig) showed no obvious difference between controls and *eAdora2b*^−/−^ mice at 2 months or 6 months of age. Next, we used a quantification method that determines the percentage area of positive antibody signal to accurately assess the neuron and astrocyte differences. The results (S2D–S2G Fig) revealed similar numbers and distribution of neurons and astrocytes between controls and *eAdora2b*^−/−^ mice at 2 months and 6 months of age. Finally, we evaluated cell death by cleaved caspase-3 (S2H Fig) and found that it was difficult to observe any positive signal in all 4 groups, indicating a low level of a cell death. Thus, ablation of erythrocyte ADORA2B does not cause obvious changes in the death of neurons and astrocytes in the CTX or HIP.

**Fig 2:**
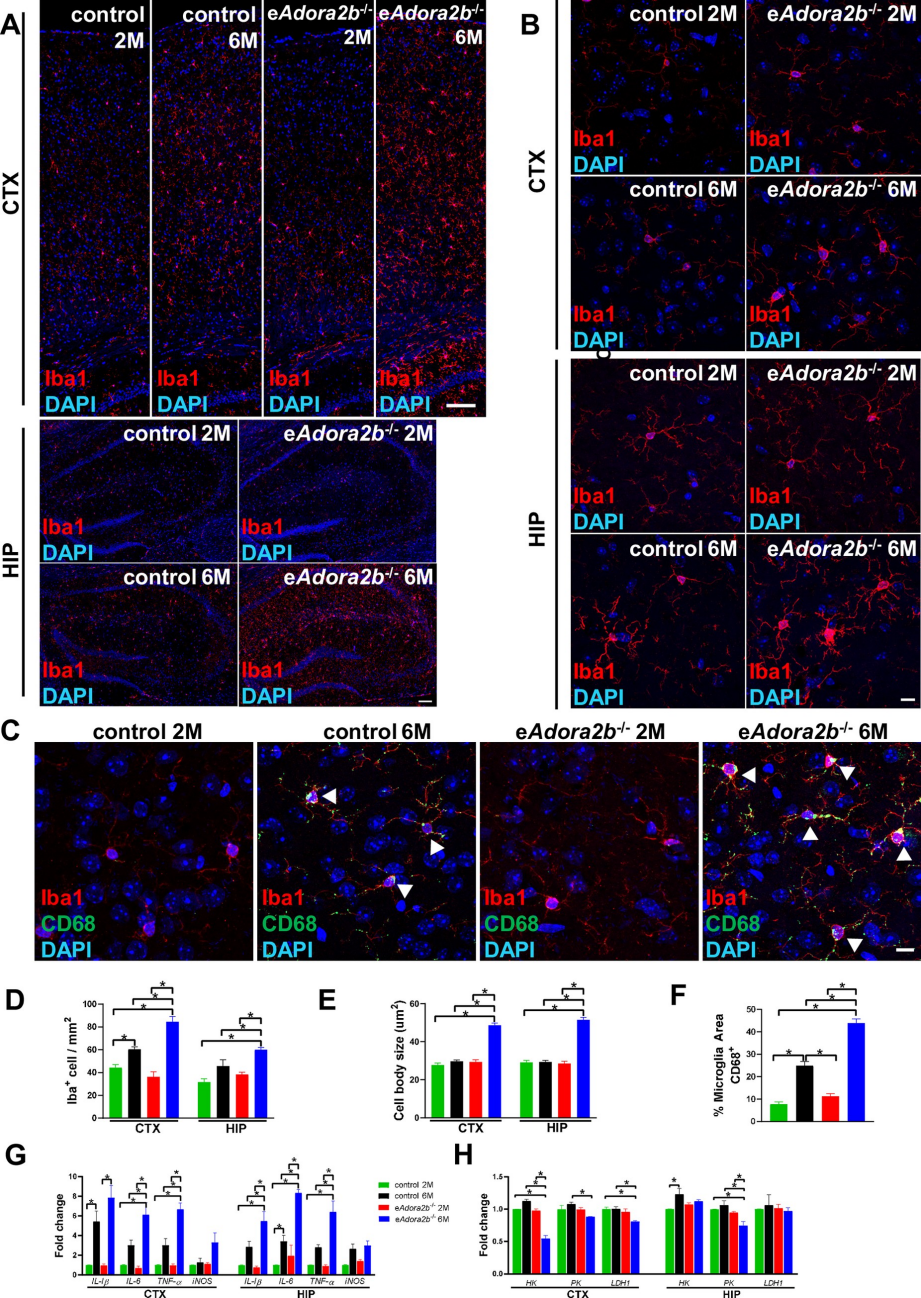
Increased abundance of activated microglia and pro-inflammatory cytokines and reduced glycolysis in brain are associated with e*Adora2b*^−/−^ mice at 6 months old. **(A)** Representative images of microglia visualized by Iba1 staining in CTX (upper) and HIP (bottom) are shown. Scale bar, 100 μm. **(B)** Representative high-magnification images show morphology of microglia in CTX and HIP. Scale bar, 10 μm. **(C)** Representative high-magnification images of activated microglia in brain by co-staining with Iba1 and CD68. Scale bar, 10 μm. Arrowheads indicate areas of co-staining. **(D)** Quantification of microglia cell count in the CTX and HIP were determined from 5 randomly selected fields for each brain region and for each of the 4 categories of mice. Data are expressed as mean ± SEM. *n* = 5 mice/group. *P < 0.05. **(E)** Morphology of microglia was assessed by measuring their cell body size from 10 cells for each brain region for each of the 4 groups of mice. Data are expressed as mean ± SEM. *n* = 5 mice/group. *P < 0.05. **(F)** Microglia activation was evaluated by assessing the proportion of Iba1-positive cells with CD68 staining from 3 randomly selected fields in the CTX and HIP for each mouse. Data are expressed as mean ± SEM. *n* = 5 mice/group. *P < 0.05. **(G)** Pro-inflammatory cytokines mRNA expression levels in CTX and HIP were measured by qRT-PCR, which were normalized to *β-actin* mRNA. Data are expressed as mean ± SEM. *n* = 4–5 mice/group. *P < 0.05. **(H)** Glycolytic enzyme mRNA expression level in CTX and HIP were measured by qRT-PCR, which were normalized to *β-actin* mRNA. Data are expressed as mean ± SEM. *n* = 3 mice/group. *P < 0.05. *P were measured by 1-way ANOVA with Tukey multiple comparison test. For all graphs, numerical data underlying plots are provided in S1 Data. ADORA2B, adenosine A2B receptor; CTX, cerebral cortex; HIP, hippocampus; IL, interleukin; iNOS, inducible nitric oxide synthase; qRT-PCR, quantitative RT-PCR; TNF-α, tumor necrosis factor alpha.

Microglia are resident macrophages that serve as antigen-presenting cells and provide a neuroinflammatory response in the central nervous system (CNS) [32,33]. As another important function of glial cells is to maintain homeostasis of the CNS, we next assessed microglia numbers and morphological changes by Iba1 antibody staining (Fig 2A). We quantified the density of microglia both in CTX and HIP (Fig 2D). Elevated number of these cells were found in the 6-month-old group, and, specifically, e*Adora2b*^−/−^ mice at 6 months presented with a higher microglia cell density than age-matched controls. Compared to the other 3 groups (Fig 2B), microglia at 6-month-old e*Adora2b*^−/−^ mice displayed less ramified morphology and were identified with a larger cell body size by quantification evaluation (Fig 2E), which is referred to as a reactive-like morphology [34]. To further confirm that microglia were in an activated state, we quantified CD68 expression in microglia, which is widely used as a marker for reactive microglia. CD68-positive microglia were difficult to detect in both controls and e*Adora2b*^−/−^ at 2 months old, but were mildly elevated in the 6-month-old controls and further significantly increased in 6-month-old e*Adora2b*^−/−^ mice (Fig 2C). In addition, quantification of reactive microglia by CD68 co-stained with Iba1 also indicated increased microglia activation in the 6-month-old e*Adora2b*^−/−^ mice (Fig 2F).

Microglia mediate the innate immune response and generate inflammatory cytokines when encountering stimuli. Importantly, a wide range of research provides evidence of links among elevated pro-inflammatory cytokines and reduced cognitive function in injury, aging, neurodegenerative disease, and other diseases [30,35]. To address the pro-inflammatory effect of microglia changes caused by genetic ablation of erythrocyte ADORA2B, we evaluated a series of pro-inflammatory cytokines in the CTX and HIP among the 4 groups. We observed a range of induction of mRNAs of cytokines such as interleukin (*IL*)*-1β*, *IL-6*, and tumor necrosis factor alpha (*TNF-α*) throughout the CTX and HIP in the controls at 6 months compared to controls at 2 months of age and a significant induction of these cytokines in the 6-month-old e*Adora2b*^−/−^ mice in comparison to 6-month-old controls (Fig 2G). These findings indicate that ablation of erythrocyte ADORA2B contributes to increased activation of microglia and inflammation in CTX and HIP of the older mice.

HIF1α mediated transcriptional regulation serves as a major adaptive response to combat hypoxia. However, attenuation of HIF1α-induced targeted genes involved in glycolysis, angiogenesis, and iron metabolism with aging could result in the impaired ability to counteract hypoxia in mice [36]. Similarly, glycolysis in the brain falls with normal aging in humans [37,38]. Thus, we evaluated a series of glycolytic enzyme expression levels from cortex and HIP. We found that a significant reduction of *HK*, *PK*, and *LDH1* in the cortex and *HK* in the HIP from 6-month-old e*Adora2b*^−/−^ mice when compared to age-matched controls. Notably, the *HK* expression level in the cortex was profoundly affected by ablation of erythrocyte ADORA2B when compared to the controls (Fig 2H). These findings demonstrated that deletion of erythrocyte ADORA2B mimics the normal aged brain with an attenuated hypoxic adaptive response and down-regulation of glycolytic enzymes in the cortex and HIP.

**Fig 3:**
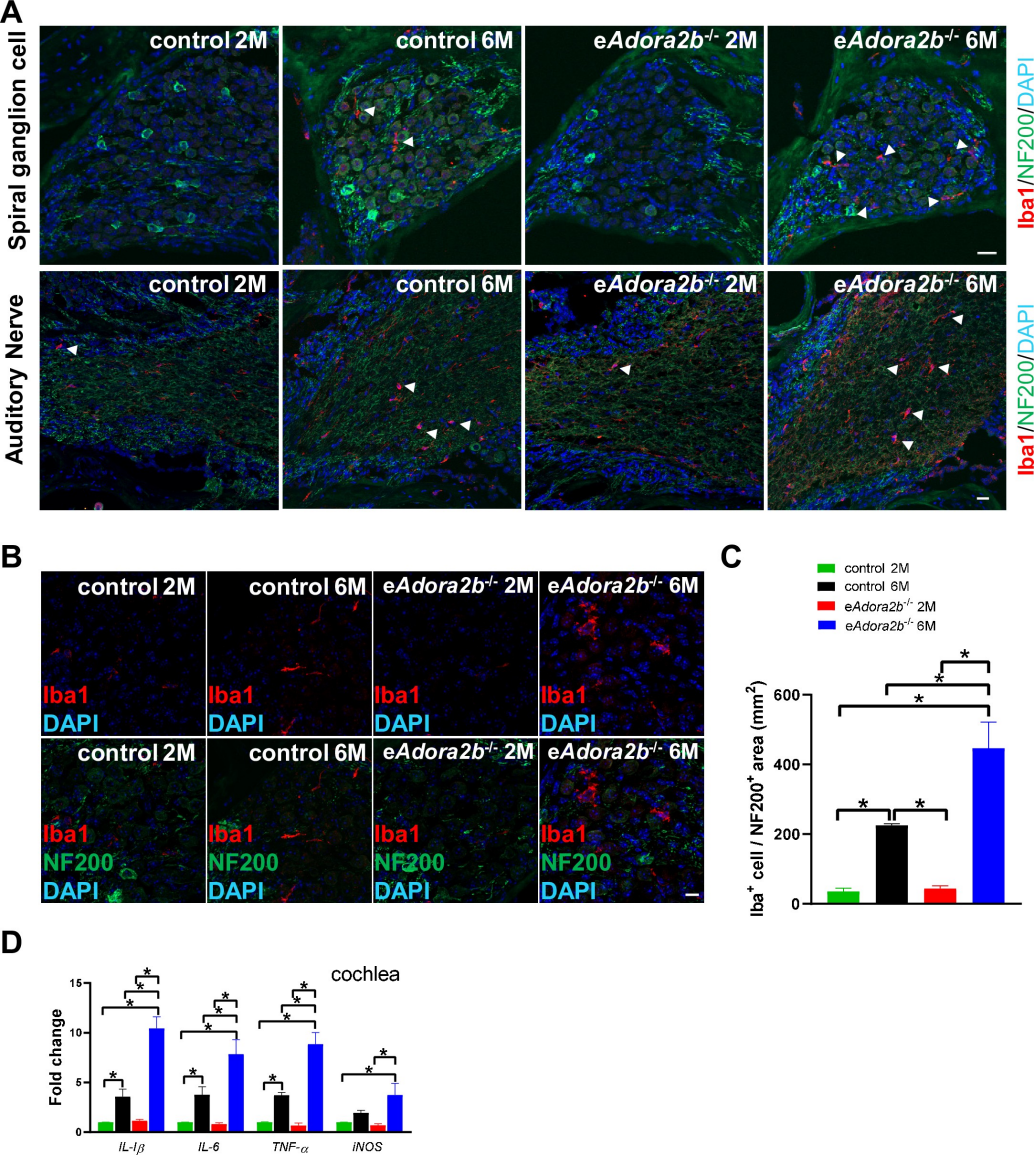
Increased abundance of enlarged macrophages and pro-inflammatory cytokines in cochlea of e*Adora2b*^−/−^ mice at 6 months old. **(A)** Representative images of macrophages around spiral ganglion cells and AN were identified by Iba1 and NF200 staining. Scale bar, 20 μm. Arrowheads indicate macrophage cells. **(B)** Representative high-magnification images show morphology of macrophages in RC. Scale bar, 10 μm. **(C)** Macrophage cells in RC were counted from apex to basal in 2 slices for each mouse. Data are expressed as mean ± SEM. *n* = 3 mice/group. *P < 0.05. **(D)** Pro-inflammatory cytokine mRNA expression levels in cochlea were measured by qRT-PCR, which were normalized to *β-actin* mRNA. Data are expressed as mean ± SEM. *n* = 4–5 mice/group, *P < 0.05. *P were measured by 1-way ANOVA with Tukey multiple comparison test. For all graphs, numerical data underlying plots are provided in S1 Data. ADORA2B, adenosine A2B receptor; AN, auditory nerve; IL, interleukin; iNOS, inducible nitric oxide synthase; qRT-PCR, quantitative RT-PCR; RC, Rosenthal’s canal; TNF-α, tumor necrosis factor alpha.

Previous studies reported that macrophages are present in the cochlea, including stria vascularis, spiral ligament, neural regions, and basilar membrane [39–42]. Macrophages are believed to contribute to myelin formation, which is important to the speed of electrical neuronal transmission [43,44]. Considering the results of the ABR tests, which refer to the region of spiral ganglion cells and AN, we targeted these 2 structures in cochlea. Iba1-positive macrophages displayed enlarged cell body size in the two 6-month-old groups and presented a variable interaction with the NF-200 positive region, which refers to spiral ganglion cells and their axons gradually bundle together to form ANs (Fig 3A and 3B). Quantification of Iba1-positive macrophage density revealed significantly increased numbers in 6-month-old e*Adora2b*^−/−^ mice compared to controls (Fig 3C). Meanwhile, 6-month-old control mice also showed an increased number of macrophage cells relative to 2-month-old mice, which may contribute to the HL that occurs at 6 months on the C57BL/6 background.

Similar to microglia in brain, macrophages in cochlea also serve as an important role in immune responses, including the release pro-inflammatory cytokines potentially relevant to HL [45]. We analyzed cytokine expression levels in cochlea (Fig 3D). mRNA levels encoding *IL-1β*, *IL-6*, *TNF-α*, and inducible nitric oxide synthase (*iNOS*) were slightly increased in 6-month-old controls compared to 2-month-old controls, and their levels were further elevated in 6-month-old e*Adora2b*^−/−^ mice compared to 6-month-old controls. Altogether, we provide genetic evidence that erythrocyte ADORA2B is essential to prevent early onset of cognitive and cochlear functional decline by ameliorating the activation of innate immune cell and subsequent inflammatory response and maintaining the hypoxic adaptive response in brain and cochlea.

### Erythrocyte ADORA2B protects against rapid cognitive and cochlear functional decline under hypoxia

**Fig 4:**
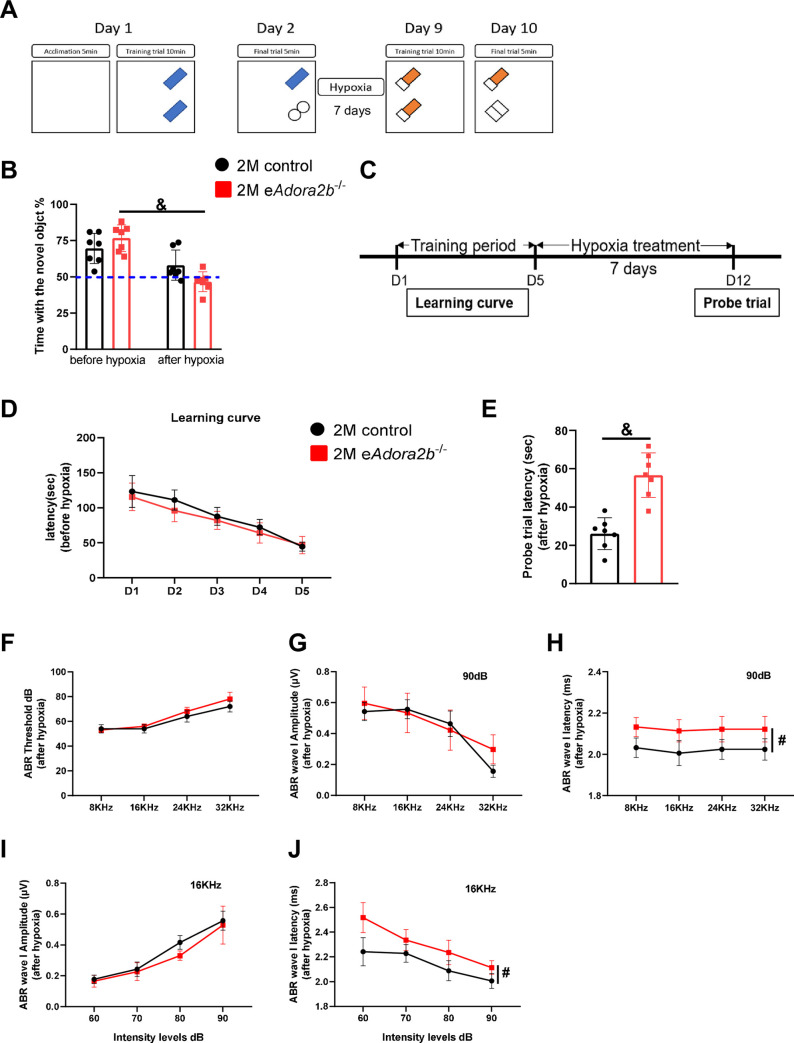
Erythrocyte ADORA2B deficiency accelerates cognitive and cochlear function decline under hypoxia. **(A)** Schematic diagram of NOR. On day 1, mice were placed in empty box with bedding for 5 minutes and then trained with 2 identical objects for 10 minutes. On day 2, final trial was done by replacing with a novel object for mice to explore for 5 minutes. With 7 days hypoxia treatment, 2 different identical objects were placed in opposite places in box to train mice on day 9. Moreover, final trial was done by replacing with a novel object on day 10. **(B)** Percentage of time exploring the novel object was quantified in final trial of NOR. A percentage lower than 50% indicates impaired cognitive abilities. Data are shown as mean ± SEM. *n* = 7 mice/group. &*P* < 0.05 were analyzed by paired *t* test. **(C)** Time course of BM. Learning curves were obtained from training period during days 1–5. After 7 days hypoxia treatment, probe trial was done on day 12. **(D, E)** Primary latency of learning curve (D) and probe trial (E) were measured. Data are presented as mean ± SEM. *n* = 7 mice/group. &*P* < 0.05 were assessed by unpaired *t* test. **(F–J)** ABR test was applied to measure HL in the 4 groups after hypoxia treatment. Threshold (F), Wave I amplitude (G) and latency (H) in response to 90 dB, and Wave I amplitude (I) and latency (J) in response to 16 KHz were measured. Data are shown as mean ± SEM. *n* = 5 mice/group. #*P* < 0.05 were measured by 2-way ANOVA with Tukey post hoc test. For all graphs, numerical data underlying plots are provided in S1 Data. ABR, auditory brainstem response; ADORA2B, adenosine A2B receptor; BM, Barnes Maze test; HL, hearing loss; NOR, novel object recognition.

Recent studies showed that erythrocyte ADORA2B-mediated hypoxia adaption is important to protect multiple tissues and organs during hypoxia exposure [23]. In view of these findings, we hypothesized that hypoxia is a driving force of the aging process and that loss of the ability to adapt to hypoxia in e*Adora2b*^−/−^ mice accelerated age-related functional decline. As presented above, there were no significant differences between e*Adora2b*^−/−^ mice and controls at 2 months of age under normoxia. Thus, we chose to expose 2-month-old controls and *eAdora2b*^−/−^ mice to hypoxia (8% O_2_) for 7 days and assess their cognitive, memory, and cochlear functions. First, we modified NOR test procedure by assessing their basal NOR at normoxia, followed by 7-day hypoxic challenge and measuring their NOR again to quantify hypoxia-mediated changes in cognitive function (Fig 4A). As a result, we found no significant difference in performance of the NOR test between 2-month-old controls and *eAdora2b*^−/−^ mice prior to hypoxia treatment (Fig 4B). However, following a 7-day exposure to hypoxia, the control mice displayed a slightly decreased interest in investigating the novel object compared to a significantly reduced NOR in e*Adora2b*^−/−^ mice (Fig 4B and 4C). Next, we assessed spatial learning and memory by the BM. Briefly, we assessed their basal learning curve at normoxia within 5 consecutive days, followed by 7-day hypoxic challenge and measuring their probe trial latency to quantify hypoxia-mediated changes in spatial learning and memory (Fig 4D). During training period before hypoxia treatment, learning curve obtained from control and e*Adora2b*^−/−^ mice showed similar results (Fig 4E). However, after 7-day exposure to hypoxia, e*Adora2b*^−/−^ mice took a much longer time to find the escape box than control group, reflecting a deficit in memory ability (Fig 4F). Thus, erythrocyte ADORA2B plays an important role to counteract hypoxia-induced cognitive, learning, and memory decline.

In the ABR test, both controls and e*Adora2b*^−/−^ mice presented similar hearing ability prior to hypoxia (S1B Fig). After 7 days hypoxia treatment, no significant differences in threshold of frequencies between controls and mutant mice were observed (Fig 4G). We then chose 90-dB intensity and 16 KHz to further analyze the ABR waveforms. The results show no significant differences between e*Adora2b*^−/−^ mice and controls in Wave I amplitude for variable frequencies and intensities following hypoxia treatment (Fig 4H and 4J). However, e*Adora2b*^−/−^ mice exhibited a longer latency of Wave I than controls in response to a range of frequencies and intensities of sound (Fig 4I and 4K). These results indicate that erythrocyte ADORA2B is required to ameliorate hypoxia-induced reduction in the transmission speed of SGC and AN. Thus, hypoxia accelerates aging-associated learning, memory, and hearing decline in 2-month-old e*Adora2b*^−/−^ mice compared to control mice.

### Erythrocyte ADORA2B is required to attenuate hypoxia-induced activation of immune cells and elevation of inflammatory response in brain and cochlea

To probe the mechanism underlying cognition and hearing decline in e*Adora2b*^−/−^ mice at structural and cellular level under hypoxia, we also performed IF staining on brain and cochlear sections. By staining for NeuN and GFAP in CTX and HIP, we found that neurons and astrocytes were distributed at similar levels in e*Adora2b*^−/−^ mice and controls following hypoxia treatment (S3A–S3C and S3E–S3H Fig). Moreover, no obvious cell death was detected in either group following hypoxia based on cleaved caspase-3 IF staining (S3D Fig).

**Fig 5:**
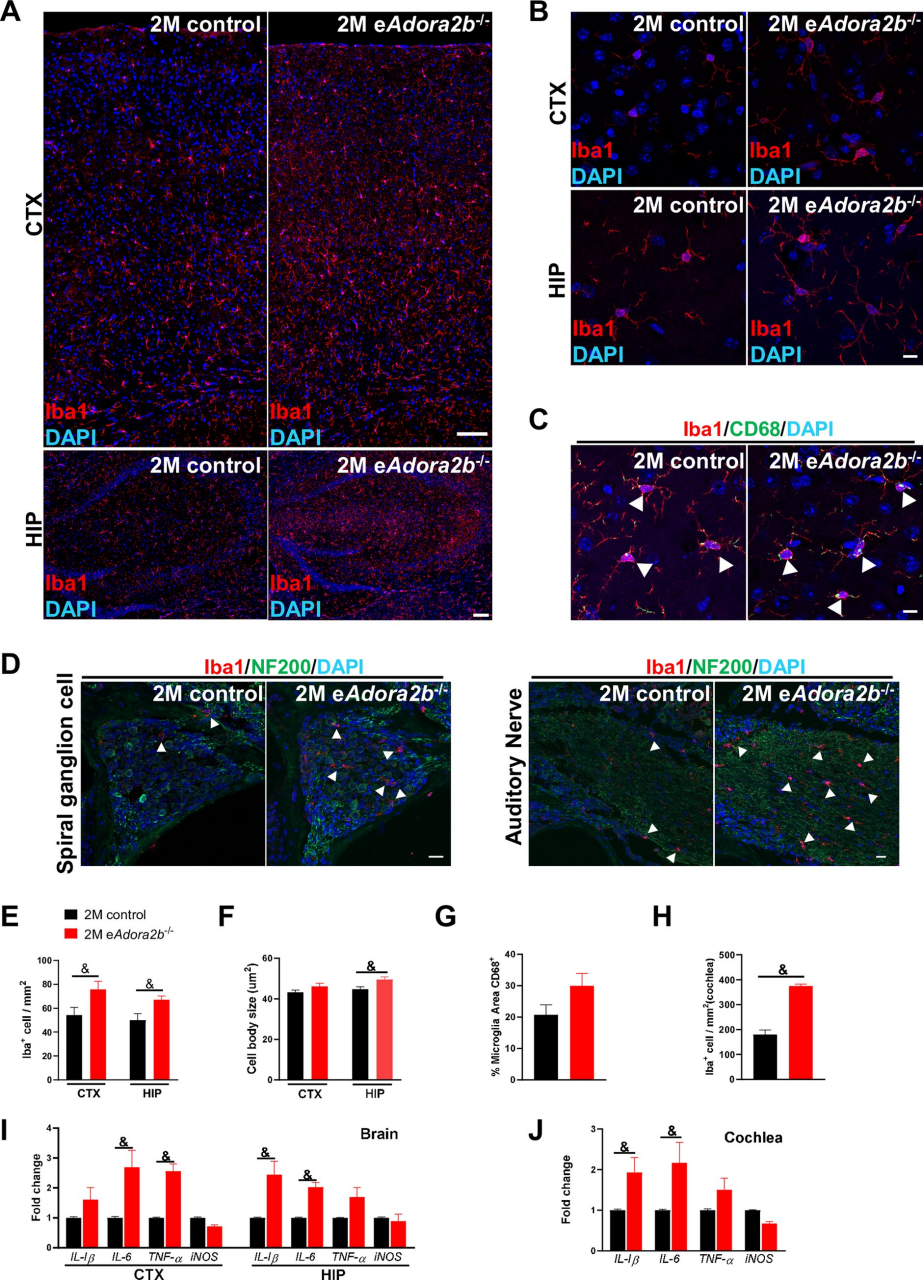
Increased abundance of activated microglia/macrophages and pro-inflammatory cytokines in tissues of e*Adora2b*^−/−^ mice following hypoxia treatment. **(A–C)** Representative images of microglia from brain. (A) Representative images of microglia visualized by Iba1 staining in CTX and HIP are shown. Scale bar, 100 μm. (B) Representative high-magnification images show morphology of microglia in CTX and HIP. Scale bar, 10 μm. (C) Representative high-magnification images of activated microglia in brain visualized by co-staining Iba1 and CD68. Scale bar, 10 μm. Arrowheads indicate areas of co-staining. **(D)** Representative images of macrophages around spiral ganglion cells and AN were identified by Iba1 and NF200 staining. Scale bar, 20 μm. Arrowheads indicate macrophage cells. **(E)** Quantification of microglia in the CTX and HIP from 5 randomly selected fields for each brain region per mouse. Data are expressed as mean ± SEM. *n* = 5 mice/group. &*P* < 0.05. **(F)** Morphology of microglia was assessed by measuring their cell body size from 10 cells for each brain region per mouse. Data are expressed as mean ± SEM. *n* = 5 mice/group. &*P* < 0.05. **(G)** Microglia activation was evaluated by assessing the proportion of Iba1-positive cells with CD68 staining from 3 randomly selected fields in CTX and HIP per mouse. Data are expressed as mean ± SEM. *n* = 5 mice/group. &*P* < 0.05. **(H)** Number of macrophages around NF200^+^ SGN cells were counted from apex to basal in 2 slices for each mouse. Data are expressed as mean ± SEM. *n* = 3 mice/group. &*P* < 0.05. **(I)** Pro-inflammatory cytokine mRNA expression levels in CTX and HIP were measured by qRT-PCR, which were normalized to *β-actin* mRNA. Data are expressed as mean ± SEM. *n* = 5 mice/group, &*P* < 0.05. **(J)** Pro-inflammatory cytokine mRNA expression levels in cochlea were measured by qRT-PCR and were normalized to β-actin mRNA. Data are expressed as mean ± SEM. *n* = 5 mice/group, &*P* < 0.05. &*P* were assessed by unpaired *t* test. For all graphs, numerical data underlying plots are provided in S1 Data. ADORA2B, adenosine A2B receptor; CTX, cerebral cortex; HIP, hippocampus; IL, interleukin; iNOS, inducible nitric oxide synthase; qRT-PCR, quantitative RT-PCR; TNF-α, tumor necrosis factor alpha.

Next, we quantified microglia and assessed their activation by IF staining for Iba1 in brain following hypoxia (Fig 5A and 5B). In e*Adora2b*^−/−^ mice, a higher density of IF staining of Iba1 (Fig 5E) throughout CTX and HIP of microglia was observed, while a larger cell body size (Fig 5F) was observed only in the HIP but not in the CTX. Meanwhile, double-positive staining of Iba1 and CD68 showed a trend toward microglia activation (without significance) in e*Adora2b*^−/−^ mice (Fig 5C and 5G). Similarly, we found that e*Adora2b*^−/−^ mice presented more macrophages surrounding NF200^+^ neuronal area and along their axon in cochlea following hypoxia treatment (Fig 5D and 5H). Thus, erythrocyte ADORA2B is essential to mitigate hypoxia-induced activation of immune cells in the brain and cochlea.

Finally, quantitative RT-PCR (qRT-PCR) analysis showed that in the cerebral CTX, there was a significant increase in *IL-6* and *TNF-α* mRNA levels and an elevated trend but lack of significance of *IL-1β* in e*Adora2b*^−/−^ mice following hypoxia treatment. In the HIP and cochlea, *IL-1β* and *IL-6* mRNA expression showed significant elevation, while *TNF-α* mRNA expression was also higher in e*Adora2b*^−/−^ mice than controls under hypoxia, but the increase did not reach a significant level. Among 3 regions (CTX, HIP, and cochlea), *iNOS* mRNA levels presented little differences between controls and e*Adora2b*^−/−^ mice under hypoxia, indicating that this gene was not specifically regulated by impact of genetic ablation of erythrocyte ADORA2B under hypoxia (Fig 5I and 5J). Altogether, we revealed that erythrocyte ADORA2B plays a previously unrecognized beneficial role to attenuate hypoxia-induced immune response and cognitive and cochlear functional decline.

### Erythrocyte ADORA2B-mediated activation of BPG mutase and the induction of 2,3-BPG production is a common compensatory mechanism to protect against aging and hypoxia-induced cognitive and cochlear functional decline

**Fig 6:**
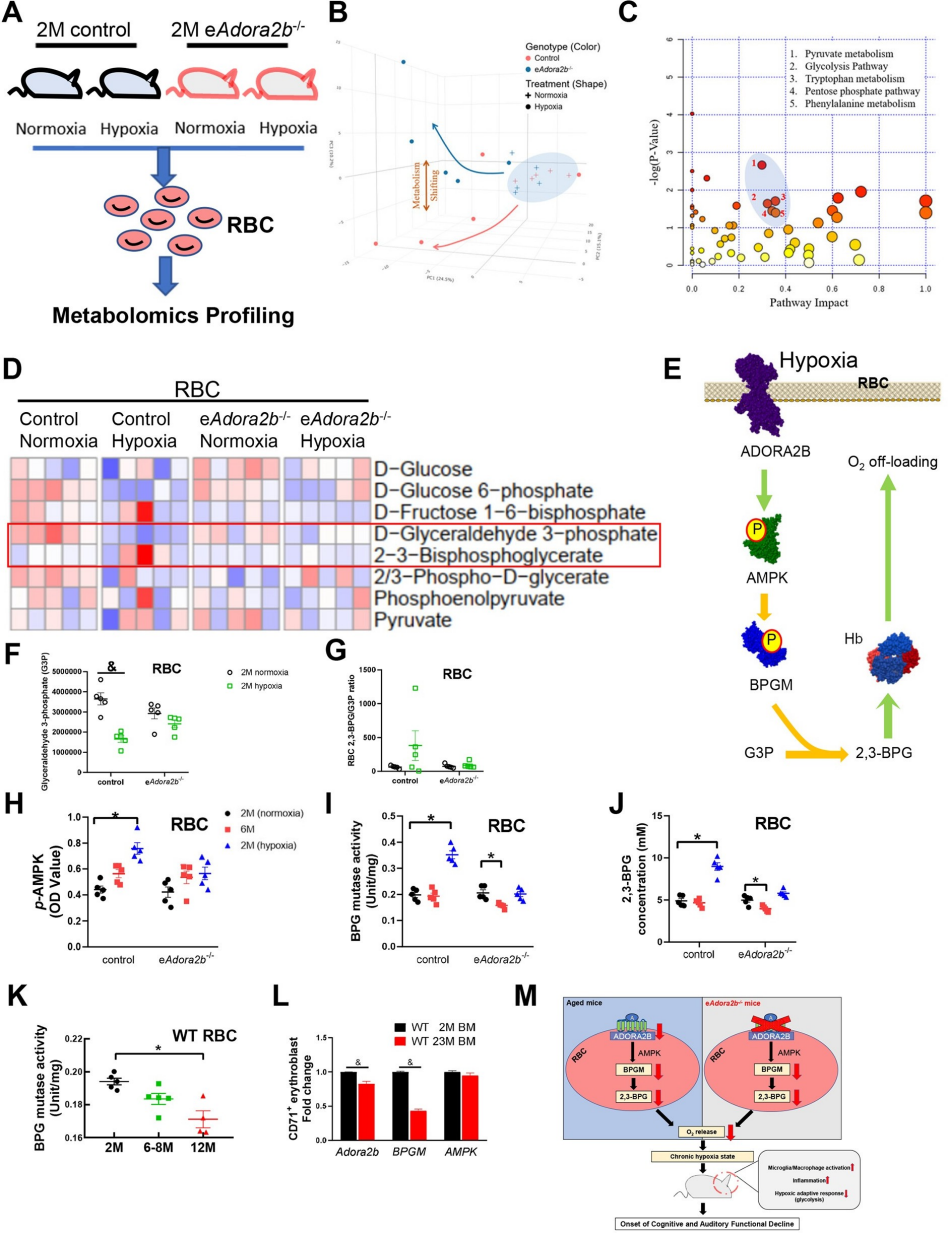
Hypoxia as the driving force resulted in reduced erythrocyte-mediated hypoxic metabolic reprogramming during aging. **(A)** Schematic representation of experimental design. Erythrocytes collected from 2-month-old controls and e*Adora2b*^−/−^ mice with or without hypoxia treatment were evaluated by unsupervised metabolomics. *n* = 5 mice/group. **(B)** PCA revealed metabolic shift of e*Adora2b*^−/−^ mice compared to control mice with or without hypoxia. **(C)** Metabolic pathway impact score of e*Adora2b*^−/−-^ and control mice with or without hypoxia treatment. **(D)** Heatmap of erythrocyte glucose metabolism measured by unbiased metabolomics profiling. **(E)** Schematic graph illustrating erythrocyte ADORA2B–AMPK–BPGM signaling pathway to promote G3P metabolized toward 2,3-BPG and thus induce more O_2_ release to counteract tissue hypoxia. **(F, G)** G3P level and (F) an 2,3-BPG/G3P ratio (G) in Metabolomic screening. Data are expressed as mean ± SEM. *n* = 5 mice/group. &*P* < 0.05 were measured by unpaired *t* test. **(H)** Erythrocyte AMPK 1α activity was measured by commercial ELISA kit. Data are expressed as mean ± SEM. *n* = 5 mice/group. *P < 0.05. **(I–K)** Erythrocyte BPGM activity (I, K) and 2,3-BPG (J) was measured by commercial kits. Data are expressed as mean ± SEM. *n* = 4–5 mice/group. *P < 0.05. **(L)** *Adora2b*-*AMPK*–*BPGM* axis mRNA expression level in CD71^+^ erythroblasts isolated from bone marrow were measured by qRT-PCR and were normalized to *β-actin* mRNA. Data are expressed as mean ± SEM. *n* = 3 mice/group, &*P* < 0.05. &*P* were assessed by unpaired *t* test. **(M)** Graphical abstract. *P were assessed by 1-way ANOVA with Tukey multiple comparison test. For all graphs, numerical data underlying plots are provided in S1 Data. 2,3-BPG, 2,3-bisphosphoglycerate; ADORA2B, adenosine A2B receptor; AMPK, AMP-activated protein kinase; BPGM, bisphosphoglycerate mutase; G3P, glyceraldehyde 3-phosphate; PCA, principal component analysis; qRT-PCR, quantitative RT-PCR; RBC, red blood cell; WT, wild-type.

Here, we provide proof-of-principle genetic evidence for the beneficial role of erythrocyte ADORA2B in attenuating cognitive and hearing functional decline under hypoxia as well as during the normal aging process. In an effort to determine the molecular and metabolic basis underlying erythrocyte ADORA2B-mediated protection from hypoxia-induced cognitive and hearing functional decline, we conducted unsupervised high-throughput metabolomics to determine the metabolic changes in erythrocytes isolated from 2-month-old controls and e*Adora2b*^−/−^ under normoxia and after 7 days hypoxia treatment (8% O_2_) (Fig 6A). A total of 222 metabolites were identified (S4 Fig, S1 Table). With principal component analysis (PCA), we found a significant metabolic shift following hypoxia in contrast to a lack of metabolic differences between e*Adora2b*^−/−^ and control mice under normoxia (Fig 6B). Metabolic pathway impact score analyses demonstrated that glycolysis was profoundly affected in e*Adora2b*^−/−^ mice under hypoxia compared to controls (Fig 6C). As described in a previous report [23], erythrocyte ADORA2B-mediated activation of AMPK–BPGM promotes glyceraldehyde 3-phosphate (G3P) metabaolized toward 2,3-BPG production, which results in releasing O_2_ from HGB to counteract hypoxia (Fig 6E). Among all the detected glycolytic metabolites, erythrocyte 2,3-BPG levels were induced under hypoxia in control mice, while its induction was substantially attenuated in e*Adora2b*^−/−^ mice (Fig 6D). G3P was significantly decreased under hypoxia in control, while its reduction was substantially attenuated in e*Adora2b*^−/−^ mice (Fig 6F). In the meantime, 2,3-BPG/G3P ratio were increased in a certain range under hypoxia in control mice compared to e*Adora2b*^−/−^ mice, although without statistical significance attributed to considerable variations (Fig 6G), implicating that erythrocyte ADORA2B activation is essential for hypoxia-induced glycolysis and increase 2,3-BPG production via AMPK–BPGM signaling cascade.

Previous human studies showed that erythrocyte 2,3-BPG levels were reduced during aging [25,26]. This finding and our metabolomic profiling raised an intriguing possibility that erythrocyte ADORA2B signaling–mediated AMPK and BPGM activation channeled glucose metabolism toward glycolysis resulting in increased 2,3-BPG production. Our results suggest that this is the common compensatory molecular and metabolic basis for maintaining normal aging process and attenuating hypoxia-accelerated cognitive and hearing functional decline. To test this intriguing hypothesis, we measured the activities of erythrocyte AMPK and BPGM and 2,3-BPG concentration from e*Adora2b*^−/−^ and control mice at 2 months and 6 months of age under normoxia and 2-month-old mice following 7 days hypoxia treatment (8% O_2_). AMPK activity did not show significant differences between different age groups from either controls or mutants. However, AMPK activity was significantly induced in 2-month-old control mice but not e*Adora2b*^−/−^ mice by hypoxia (Fig 6H). These results suggest that ADORA2B-mediated AMPK activation is a compensatory mechanism to counteract hypoxia-induced cognitive and hearing functional decline but not normal aging under normoxia. BPG mutase activity and 2,3-BPG concentration were maintained at the similar levels at 2 months and 6 months of age in controls under normoxia and further induced in 2-month-old control mice by hypoxia. BPG mutase activity and 2,3-BPG concentration were significantly reduced in 6-month-old e*Adora2b*^−/−^ mice under normoxia, and their induction by hypoxia was attenuated in 2-month-old e*Adora2b*^−/−^ mice (Fig 6I and 6J). Thus, these results support our conclusion that erythrocyte ADORA2B-mediated BPG mutase activation underlies erythrocyte hypoxia metabolic reprogramming toward glycolysis to induce 2,3-BPG production to protect against hypoxia and aging-mediated cognitive and hearing functional decline.

### Erythroblast ADORA2B and BPGM gene expression and erythrocyte BPGM activity are reduced with normal aging

Early studies showed that erythrocyte 2,3-BPG levels are reduced in healthy aged humans comparing to young individuals and further reduced in patients with AD [25–27]. Here, we showed that loss of erythrocyte ADOAR2B leads to decreased BPG mutase activation and 2,3-BPG production and accelerates age-related memory and hearing decline. These findings prompted us to hypothesize that erythrocyte BPG mutase activity is reduced and contributes to reduced 2,3-BPG production during normal aging. To test this possibility, we measured erythrocyte BPGM activity from young around 2 months old to aged mice up to 12 months. Intriguingly, we found that erythrocyte BPG mutase activity was slightly reduced in 8-month-old WT mice comparing to young (2 months old) WT mice, but significantly reduced in 12-month-old WT mice (Fig 6K), indicating that reduction of erythrocyte BPGM is a natural progression of normal aging.

Next, to determine whether ADORA2B, AMPK, and BPGM gene expression are already down-regulated in nucleated erythroid precursor during normal aging, we conducted RT-PCR to quantify *Adora2b*, *BPGM*, and *AMPK* mRNA expression levels in purified CD71^+^ erythroblasts isolated from bone marrow of young (2 months old) and aged (23 months old) WT mice as previous study [1]. We found that erythroblast ADORA2B and BPGM but not AMPK gene expression levels were significantly decreased with aging (Fig 6L). Thus, these results revealed that reduced ADORA2B and BPGM gene expression in erythroblasts and decreased BPGM activity in erythrocytes are natural progression of normal aging, which could lead to age-related functional decline.

## Discussion

Upon aging, age-related functional decline (including cognitive and cochlear functional decline) becomes a major health challenge worldwide, making it important to identify the specific rejuvenating factors to combat onset of aging and slow down the progression to functional decline. Hypoxia has been long speculated as a driving force for aging. Although the erythrocyte is the most abundant cell type in our body and the only cell type delivering O_2_, its function in aging and progression to age-related functional decline remains undetermined. Here, we report that erythrocyte ADORA2B is a previously unrecognized purinergic component counteracting aging at the functional, cognitive, auditory, structural, and cellular level. Functionally, we demonstrated that genetic ablation of erythrocyte-specific ADORA2B (e*Adora2b*^−/−^*)* leads to an early onset of aging process displaying cognitive and cochlear functional decline with a profound inflammatory response including elevated activation of innate immune cells and cytokines in the CTX, HIP, and cochlea at a young age. Intriguingly, we demonstrated that hypoxia directly accelerates the aging process and that erythrocyte ADORA2B signaling plays a previously unrecognized beneficial role to counteract hypoxia-exacerbated aging by maintaining cognitive and cochlear function as well as attenuating hypoxia-induced cerebral and cochlear inflammatory response. Mechanistically, we revealed that activation of the erythrocyte-specific ADORA2B–AMPK–BPGM signaling axis plays a role in cognitive and cochlear function decline and inflammatory response as a function of aging and hypoxia. In keeping with our previous studies on high-altitude hypoxia, we report that this signaling axis promotes erythrocyte hypoxic metabolic reprogramming to favor glycolysis and 2,3-BPG production, which promote HGB O_2_ off-loading and counteract hypoxia. Mimicking e*Adora2b*^−/−^ mice, we further discovered that down-regulation of BPG mutase activity in RBCs is a natural process during normal aging in WT mice. These findings led us to further discover that *Adora2b* and *BPGM* mRNA levels are already significantly reduced in CD71^+^ erythroblasts in WT aged mice comparing to young mice. These findings provide the critical support for our working model that reduced erythroblast ADORA2B–BPGM gene expression and erythrocyte BPGM activation leads to chronic hypoxia state, attenuated hypoxic-induced adaptive response including decreased glycolytic gene expression, pro-inflammatory response, and, thus, progression of memory and hearing decline during normal aging (Fig 6M). Overall, our studies indicate a critical role of erythrocyte ADOAR2B–BPGM as an “anti-aging” and “anti-hypoxia” rejuvenating factor by inducing 2,3-BPG production and triggering O_2_ delivery to attenuate progression to age-related functional decline and immediately suggest novel therapies targeting this signaling axis.

### Erythrocyte ADORA2B: Anti-aging and anti-hypoxia–induced cognitive and cochlear functional decline

Erythrocytes are very sensitive to hypoxia, and their O_2_ delivery capacity is finely regulated by metabolic control. Using human metabolomic screening and mouse genetic studies, recent research revealed a beneficial role of ADORA2B-mediated hypoxic metabolic reprogramming for adaption to high altitude by inducing 2,3-BPG production and O_2_ release from erythrocytes to counteract tissue hypoxia in young mice. Like normal individuals facing high-altitude hypoxia, there is emerging evidence indicating that age-related decline is an age-related systemic metabolic condition frequently associated with hypoxia [46]. Unlike high-altitude hypoxia in normal individuals, aging is unable to trigger an adaptive response to counter a reduction of ATP and 2,3-BPG levels in their erythrocytes of elderly individuals compared to erythrocytes of young individuals [25,26]. 2,3-BPG is an erythroid-specific glycolytic metabolite, which functions as a negative allosteric modulator to decrease HGB-O_2_ binding affinity and, in turn, increase O_2_ deliver to peripheral tissues to mitigate hypoxic and ischemic tissue damage. Thus, the reduced erythrocyte 2,3-BPG concentration in elderly individuals can lead to an insufficient delivery of O_2_ and thus promotes tissue hypoxia including but not limited to the brain. Cerebrometabolism is extremely sensitive to hypoxia because neuronal cells do not use fatty acids as an energy source. Instead, neurons in the brain and cochlear depend largely on glucose oxidization to generate sufficient ATP to maintain neuronal function and survival. Thus, even brief O_2_ deprivation can lead to significant brain dysfunction and chronic hypoxia and can ultimately result in irreversible brain injury and permanent impairment of cognition and hearing [47,48]. Here, we demonstrated that, mimicking elderly individuals, 2,3-BPG levels are significantly lowered in older mice (6 months) compared to younger mice (2 months). At 6 months of age (early-age onset), these mice develop similar phenotypes as elderly individuals characterized by the decline in learning and memory abilities and impairment in hearing, which may be attributed to the condition of chronic tissue hypoxia. This hypothesis was further tested by exposing e*Adora2b*^−/−^ mice to hypoxia. Intriguingly, we demonstrated that hypoxia-induced 2,3-BPG production in normal control mice as a compensatory response to offset hypoxia is lost in e*Adora2b*^−/−^ mice at a young age (2 months old). As such, e*Adora2b*^−/−^ mice exhibit accelerated development of cognitive, memory ability, and auditory functional decline with an elevated immune response in brain and cochlea as young as 2 months old after exposure to hypoxia. Overall, our studies support a novel but compelling working model that activation of erythrocyte ADOAR2B is a general compensatory mechanism to counteract physiological hypoxia, in particular, promoting tissue oxygenation by inducing 2,3-BPG production and O_2_ release from erythrocytes. However, in humans with aging as seen in e*Adora2b*^−/−^ mice, such a compensatory mechanism is lost with a reduction of 2,3-BPG, leading to chronic hypoxia and rapid progression to cognitive and hearing functional decline. Overall, our genetic studies have advanced our understanding of the beneficial role of erythrocyte ADORA2B in age-related cognitive and hearing functional decline by regulating 2,3-BPG production in the erythrocytes.

### Erythrocyte ADORA2B-mediated activation of AMPK and BPG mutase underlies hypoxic and metabolic reprogramming during aging

Previous studies [23,49] indicate that erythrocyte 2,3-BPG production is regulated by sophisticated molecular mechanisms and metabolic reprogramming mediated by an erythrocyte ADORA2B–AMPK–BPG mutase signaling pathway under hypoxia. Supporting this study, we found that hypoxia-induced activation of AMPK and BPGM, metabolic reprogramming with the increased glycolysis, and 2,3-BPG production are lost in the erythrocytes of e*Adora2b*^−/−^ mice at 2 months of age. Extending from hypoxia studies, we found that the activity of BPGM but not AMPK is significantly reduced in e*Adora2b*^−/−^ mice as early as 6 months old under normoxia. Although AMPK activity was not reduced in the erythrocytes of e*Adora2b*^−/−^ mice as early as 6 months old under normoxia, it is likely that AMPK activity is reduced only with a hypoxic challenge or at much older age beyond 6 months old. Loss of erythrocyte ADORA2B–BPGM axis mimicking normal aging leads to reduced erythrocyte 2,3-BPG levels and more proliferated and activated microglial/macrophages in HIP, cortex, and cochlea with an early onset of memory/hearing decline. Cytokine gene profiling indicates that loss of ADORA2B induces a series of pro-inflammatory cytokines implicating the pro-inflammatory microglia/macrophage is the major cell type proliferated and activated in HIP, cortex, and cochlea. Our findings support a new concept that aging is a chronic hypoxic challenge and that impaired adaptive metabolic reprogramming mediated by loss of the ADORA2B–AMPK–BPGM signaling cascade leads to decreased 2,3-BPG production, and insufficient O_2_ release is a previously unrecognized pathogenic component underlying early aging and rapid cognitive and hearing functional decline. Thus, maintaining or promoting the ADORA2B–AMPK–BPGM signaling cascade could offer a new therapeutic strategy for cognition and hearing by promoting 2,3-BPG production and O_2_ delivery. Supporting these therapeutic possibilities are previous reports that 5-aminoimidazole-4-carboxamide ribonucleotide (AICAR), an AMPK agonist, showed a positive role in the rescue of cognition decline [50,51]. Of note, metformin—an AMPK activator in nucleated cells with mitochondria [52]—has been under intense clinical investigation as a longevity treatment [53]. Vice versa, AMPK overexpression extends the life span of several animal models [54]. Whether the early age-related behavioral and auditory phenotype of e*Adora2b*^−/−^ could be rescued by activating AMPK–BPGM to produce more 2,3-BPG in RBCs is an important question to be investigated.

### Erythrocyte ADORA2B counteracts inflammatory responses during aging

The major focus of aging studies is on brain neuron degeneration, atrophy, and death, which is the end point. However, the etiopathology of early aging remains unknown. In recent years, a growing body of evidence has shown that inflammation is an early pathogenic factor linked with aging-associated pathologies and even accelerating progression of neurological functional decline [55]. For example, IL-1β, IL-6, and TNF-α can prompt aging-associated phenotypes and pathologies [56]. IL-6 is reported to be one of the most prominent cytokines across age-related pathologies [57]. IL-1β and TNF-α are known to be elevated across ARDs and serve as strong chronic inflammatory components. However, the causative factors inducing inflammation and subsequent development of cognitive decline and HL remain largely unidentified. Here, we demonstrated that erythrocyte-specific ablation of ADORA2B leads to an increase in the number and activation of microglia/macrophages accompanied with elevated pro-inflammatory cytokines and mediators, including IL-1β, IL-6, TNF-α, and iNOS in the brain and cochlea as early as 6 months under normoxia and as early as 2 months old under hypoxia. In contrast, there is little loss of neurons and no obvious changes of astrocytes in CTX and HIP in 6-month-old e*Adora2b*^−/−^ mice at normoxia and 2-month-old e*Adora2b*^−/−^ mice under hypoxia. Early studies have showed that microglia can modify synaptic plasticity via directly contacting with neurons [58], removing dendritic spines [59], and releasing molecules known to control neuronal function and synaptic transmission, such as nitric oxide, trophic factors, or cytokines [60]. Synaptic plasticity plays a central role in the process of learning and memory formation [61]. Thus, erythrocyte ADORA2B deficiency–induced early aging and accelerated development of hypoxia-induced learning and memory decline may be attributed to microglial-mediated impairment in synaptic plasticity in the CNS.

For auditory function, we demonstrated increased latency of Wave I of the ABR test in 6-month-old e*Adora2b*^−/−^ mice at normoxia and 2-month-old e*Adora2b*^−/−^ mice under hypoxia, implicating the defect of transmission of SGN to AN. Normal myelination is important to protect axons and provide for efficient transmission of action potential along the AN [62]. Both human and rodent studies showed that macrophages are recruited to and activated in the regions of myelination [43,63,64]. Because we observed that activated macrophages are recruited along NF200^+^ SGC and the AN, it is possible that erythrocyte ADORA2B deficiency–induced early age-dependent HL and accelerated development of hypoxia-induced auditory functional decline may be attributed to macrophage-mediated impairment in myelination around SGN and AN. Overall, our studies showed that deficiency in erythrocyte ADORA2B-mediated 2,3-BPG production reduces tissue oxygenation, increases the number and activation of microglia/macrophages, and, eventually, accelerates aging and progression of age-related functional decline. These findings add significant new insight into the molecular basis underlying reduction of erythrocyte metabolic reprograming and O_2_ release capacity in increased immune response upon aging. Our studies have opened up multiple new investigative directions including (1) how erythrocyte ADORA2B regulates microglia/macrophage proliferation and activation in early aging; and (2) how activated microglia/macrophages contribute to age-related cognitive and hearing functional decline. Although speculative at this stage, our findings substantiate the role of RBC biology in systems homeostasis [65], a model that could help to reconcile observations from endeavors as diverse as inflammaging, anemia of aging, and hypoxic metabolic reprogramming.

### Novelty and significance

Multiple lines of evidence demonstrated that injection of plasma from young mice [66,67], heterochronic parabiosis [68,69], and transplantation of young bone marrow [70] rejuvenates and improves cognitive and memory ability in aged mice. The rejuvenation has been mainly attributed to specific enzymes, chemokines, and other proteins contained in the young blood. However, erythrocytes are the main component of blood and have a unique function to deliver O_2_ to every single cell within our body. Recently, a new study revealed that hyperbaric oxygen treatments (HBOTs) have a beneficial role in counteracting aging of blood cells and even reverse the aging process in healthy aging adults [71]. These studies support our work presented here that erythrocytes have a significant role to counteract aging and that hypoxia is an underestimated key factor promoting aging and function decline. Nevertheless, a role for erythrocyte function in age-related cognition and hearing functional decline has not been recognized prior to the study presented here. Our findings regarding the importance of erythrocyte ADORA2B-mediated activation of AMPK and BPGM, metabolic reprogramming, and 2,3-BPG production in anti-aging and anti-age–related functional decline have advanced our understanding and provide novel molecular insight concerning the etiopathology of age-related functional decline. Significantly, our mouse genetic studies have identified a role for purinergic signaling (including ADORA2B and potentially expandable to other players in this pathway, such as equilibrative nucleoside transporter 1) in the early onset of aging. Our findings reveal novel promising circulating pathogenic biomarkers and candidate therapeutic targets in the mature erythrocyte to counteract early aging and age-related impaired cognition and hearing function—a mechanistic finding that demands further validation in humans.

## Materials and methods

### Experimental model and subject details

Erythrocyte-specific deletion of ADORA2B mice were generated by crossing mice homozygous for floxed *Adora2b* allele with mice expressing Cre recombinase under the control of the erythropoietin receptor (EpoR) gene regulatory elements [23,72]. C57BL/6 WT mice were previously purchased from Harlan Laboratories (Indianapolis, Indiana, United States of America). Mice were bred in a pathogen-free, temperature- and humidity-controlled facility on a 12-hour light and dark cycle under normoxia or hypoxia (8% O_2_) for the experiment purposes. Both water and food were supplied ad libitum. All procedures involving animals were reviewed and approved by the Institutional Animal Welfare Committee of the University of Texas Health Science Center at Houston (AWC-17-0073), and all experimental protocols were conducted according to the National Institutes of Health Guide for Care and Use of Laboratory Animals. Moreover, 2-month-old, 6-month-old, 8-month-old, 12-month-old, and 23-month-old healthy mice of both genders with required genotype were randomly assigned to experimental groups. Animals with sickness were excluded in the study. Mice were labeled with ear tag at weaning, and all researchers were blinded in the experimental procedures.

### Behavioral testing

#### Novel object recognition

Mice were placed in an open-topped, clear Plexiglass chamber with embedding and divided to 4 quadrants (Q1, Q2, Q3, and Q4). Trials were recorded, and tracking was analyzed by TopScan 2.0 (CleverSys). Mice were free to explore the environment without any positive or negative stimulus. On day 1, each mouse was initially placed in the empty chamber and freely explored the arena without objects for 10 minutes. The animal was then removed from the arena to its cage. After all the mice completed their exploring procedure, the mice started the training procedure in which each mouse was placed in the arena with 2 identical objects (Q2Block + Q3Block) for 5 minutes. On day 2, final trials were done by placing mice in the arena exposed to one of the previous objects and a novel object (Q2Block + Q3Circle) for 5 minutes. For 7 days hypoxia treatment group (Fig 4A), mice completed day 1 and day 2 trials and were placed in 8% O_2_ hypoxia chamber controlled by monitor (Okolab, Pozzuoli, NA, Italy) for 7 days. On day 9, mice were placed in the arena with 2 different identical objects, which were placed in the opposite quadrant to the previous trials for 5 minutes (Q1Pill bottle + Q4 Pill bottle). On day 10, final trials were done by placing mice in the arena with replacing a novel object (Q1Pill bottle + Q4Cap) for 5 minutes. Time with the novel object was obtained from final trial and is expressed as the time spent exploring the novel object divided by the total time exploring both the novel and familiar object multiplied by 100. Time with the novel object lower than 50% demonstrated impaired nonspatial learning and memory ability.

#### Barnes maze

The BM were performed as previously described [73,74]. A circular platform with 40 holes and surrounded by visual clues was set as the arena. One stationary escape box was set on one unchanged hole for mice to navigate by clues to exit with negative stimuli (buzz and light). On day 1, each mouse was placed in the area and guided by the researcher to the escape box without negative stimuli for familiarization procedure. Next, 2 rounds of the adaption procedure were done, which were the same procedures as familiarization procedure but with negative stimuli. Two rounds of acquisition trials were completed by placing the mice in the arena and letting them exploring freely to find the exit for no more than 3 minutes. During days 2 to 5, 4 acquisition trials were completed. Short-term memory test was designated as the first trial on day 5. Days 1 to 5 trials were termed as training period. Then, after 7 days rest, mice were assessed for long-term memory test, which is the same procedure as acquisition trial. For hypoxia treatment group (Fig 4C), each mouse was followed the same training period as described above. Next, mice were placed in hypoxia chamber (8% O_2_) for 7 days. After that, mice were assessed for probe trial on day 12. Primary latency to the escape box was recorded by TopScan system and used to assess spatial learning and memory ability. Learning curve was obtained by averaging acquisition trials’ primary latency from each day of training period. Behavioral testing was performed by a designated researcher blinded to mice genotype and experimental manipulation (normoxia versus hypoxia treatment).

### Hearing test

ABR was recorded for hearing test as previously described [75,76]. Mice were intraperitoneally injected with a mixture of ketamine (100 mg/kg) and xylazine (10 mg/kg) for anesthesia. Hearing tests were performed in a soundproof room. During the test, mice were placed on a heating pad to maintain body temperature. Generated by System 3 digital signal processing hardware and software (Tucker-Davis Technologies [TDT], Alachua, FL, USA), pure tone bursts (0.1 ms rise/fall, 2 ms duration, and 21 presentations/s) from 8 to 32 KHz stimulated mice to arouse response. The intensity of tone stimuli was adjusted using a type 4937 one-quarter inch pressure-field calibration microphone (Brüel & Kjær, Nærum, Denmark). Response signals were received by 3 electrodes, which were subcutaneously inserted at the vertex of the scalp (channel 1), the postauricular bulla region (references), and the back leg (ground) and averaged over 512 presentations of the tone bursts. ABR waveforms were recorded in 5-dB intervals followed from the initial 90-dB amplitude until the waveforms were hardly distinguished. Threshold was defined as lowest stimuli to clearly arouse peaks of Waves I to V. To compare Wave I amplitude and latency, we used TDT system software to analyze. Amplitude was measured by the value of the peak of the waveform subtracting the troughs, and latency was calculated as the time from the start of stimulus to onset of the waveform.

### Histological analysis

Mice were anesthetized with 2.5% Avertin, blood was withdrawn transcardially, and then perfused with PBS. Mouse cochlea and brain were harvested and postfixed overnight in 4% PFA solution, followed by cryoprotection in 30% sucrose in PBS for at least 3 days. Cochlea was decalcified in 5% EDTA solutions after postfixed for 3 days and then put in 30% sucrose in PBS. After embedding in Tissue-Tek optimal temperature compound (Sakura Finetek, Torrance, CA, USA), 10-μm sections of brain and cochlea were prepared on a cryostat and mounted on slides and stored at −20°C. Brain coronal sections from each mouse were selected with similar anatomical locations near bregma −2 mm based on mouse brain atlas from each mouse. Frozen sections were washed with PBS, then blocked and permeabilized with 0.3% Triton X-100 (Sigma, St. Louis, MO, USA) and 10% BSA (Sigma) in PBS at room temperature (RT) for 2 hours. Sections were incubated with primary antibodies diluted with blocking buffer (0.3% Triton X-100 and 10% BSA in PBS) overnight at 4°C. With 5-minute washing in PBS for 3 times, sections were incubated with secondary antibodies diluted with blocking buffer for 1 hour at RT. After 3 times 5-minute washing, sections were mounted with ProLong Gold Antifade Mountant with DAPI (Thermo Fisher Scientific, Cat# P36935, Waltham, MA, USA). Primary antibodies used are the following: anti-NeuN (Abcam, Cat# ab104225, 1:1,000, Cambridge, UK), anti-GFAP (Invitrogen, Cat#Pa1-10019, 1:500, Waltham, MA, USA), anti-activated caspase-3 (Cell Signaling Technology, Cat#9661, 1:500 Danvers, MA, USA), anti-Iba1 (Wako, Cat#019–19741, 1:500, Osaka, Japan), anti-CD68 (Bio-Rad, Cat#MCA1957GA, 1:200, Hercules, CA, USA), and anti-NF200 (MilliporeSigma, Cat#N0142, 1:300, St. Louis, MO, USA). Secondary antibodies included anti-rabbit Alexa Fluor 594 (Thermo Fisher Scientific, Cat#A11012, 1:1,000), anti-rat Alexa Fluor 488 (Thermo Fisher Scientific, Cat#A21208, 1:1,000), and anti-mouse Alexa Fluor 488 (Thermo Fisher Scientific, Cat#A21202, 1:1,000).

Images were captured with Zeiss LSM 880 confocal microscope and processed with Zen blue edition lite 3.0 (Carl Zeiss, Oberkochen, Germany) to generate maximum intensity projections of each image. Images were quantified by Zen blue edition lite or ImageJ software. For NeuN and GFAP, to quantify neurons and astrocytes in the cortex and HIP, images were acquired using the tiles and positions module with z-stack by a 20× objective lens. Projection images were imported to ImageJ and equal threshold setting applied to calculate positive area of certain antibody divided by total structure area to get a percentage area. For the quantification of microglia density, 5 randomly selected fields were captured by 20× objective lens and counted for Iba1-positive cells per mouse. Average number of microglia from 5 fields was divided by total picture area to determine density. Representative images of cortex and HIP were acquired using the tiles and positions module with z-stack by a 20× objective lens. For morphology of microglia, images were acquired by 63× oil objective lens. Cell body size were collected from 10 cells per mice and measured by using the Draw Spline Contour in the Zen software. For CD68, images taken by 63× oil objective lens from 3 randomly selected fields were imported to ImageJ. CD68-positive area was divided by Iba1-positive area to determine percentage area for quantification. For density of macrophage around NF200^+^ SGN cells in Rosenthal’s canal, images taken by 20× objective lens from 2 slices per mice were averaged counting for Iba1-positive cells. Average number of macrophages was divided by the area of Rosenthal’s canal to determine macrophage density. Quantification was performed by a designated researcher blinded to mice genotype.

### Collection of CD71^+^ erythroid progenitor cells from bone marrow

Bone marrow cells (BMCs) were collected by flushing femur and tibias with PBS using syringe from designated mice. Next, BMCs were centrifuge at 300 g for 10 minutes, and supernatant was removed. BMCs were resuspended in 90-μl PBS buffer (PBS containing 0.5% BSA), and 10-μl rat anti-mouse CD71 antibody (Santa Cruz Biotechnology, Cat#59112, Dallas, TX, USA) was added to the buffer. BMCs were incubated with antibody on ice for 60 minutes. CD71 conjugated BMCs were washed with PBS twice and then resuspended in 500-μl PBS buffer with 20-μl goat anti-rat IgG magnetic beads (New England Biolabs, Cat#S1433S, Ipswich, MA, USA). Mixture was incubated at 4°C for 30 minutes. Magnet was applied to positive-select CD71^+^ erythroid progenitor cells (erythroblasts) from BMCs and stored cells at −80°C.

### Quantitative RT-PCR

Mice were anesthetized with 2.5% Avertin, and blood was withdrawn transcardially. Organs were harvested and frozen in liquid nitrogen, then stored in −80°C. RNA was extracted from stored organs or cells by TRIzol (Invitrogen) method with or without using VWR 200 homogenizer (VWR, Radnor, PA, USA). We used QuantiTect Reverse Transcription Kit (Qiagen, Germantown, MD, USA) to generate cDNA. qRT-PCR was performed using QuantiFast SYBR Green PCR kit (Qiagen) on an LightCycler 480 Real-Time PCR system (Roche, Basel, Switzerland). The primer pairs were used as follows: *β-Actin* forward 5′-GTGACGTTGACATCCGTAAAGA-3′ and reverse 5′-GCCGGACTCATCGTACTCCC-3′; *IL-6* forward 5′-CTGCAAGAGACTTCCATCCAG-3′ and reverse 5′-AGTGGTATAGACAGGTCTGTTGG-3′; *IL-1β* forward 5′-CGCAGCAGCACATCAACAAG-3′ and reverse 5′-GTGCTCATGTCCTCATCCTG-3′; *TNF-α* forward 5′-CAGGCGGTGCCTATGTCTC-3′ and reverse 5′-CGATCACCCCGAAGTTCAGTAG-3′; *iNOS* forward 5′-TGGAGCGAGTTGTGGATTGTC-3′ and reverse 5′-CCAGTAGCTGCCGCTCTCAT-3′; *Adora2b* forward 5′-GCATCGCGAATAAAAGCTGC-3′ and reverse 5′-ATGAGCAGTGGAGGAAGGAC-3′; *BPGM* forward 5′-GAGCGTCACTATGGAGCCTT-3′ and reverse 5′-AGTGGGCAGAGTGATGTTGA-3′; *AMPK* forward 5′-AGATCGGCCACTACATCCTG-3′ and reverse 5′-CGAATCTTCTGCCGGTTGAG-3′; *HK* forward 5′-TCAAAGAGAACAAGGGCGAGG-3′ and reverse 5′-GAGGAAGCGGACATCACAATC-3′; *PK* forward 5′-CGTCCGCAGGTTTGATGAGA-3′ and reverse 5′-GATGACAGGCTTCCCAGCTC-3′; *LDH1* forward 5′-TCAGTCGCCCAAGGTTATGG-3′ and reverse 5′-CAGCCTCTGCTTCTACCGTC-3′. The level of mRNA expression was normalized to *β-Actin* expression, and relative mRNA expression was calculated by 2^−ΔΔCt^ method. For each mRNA expression analysis, *n* = 5 mice per group were used.

### Complete blood cell counts and erythrocyte 2,3-BPG

After transcardial blood withdraw from mice, 60 μl were collected into heparinized tubes for automated complete blood cell counts (CLAMC of UThealth, Houston, Texas, USA). Moreover, 20-μl blood were mixed with 100-μl 0.6N cold perchloric acid preserved (PCA) on ice for at least 10 minutes. The mixture was centrifuged at 13,000 rpm for 10 minutes. Moreover, 80-μl supernatant was transferred to a new tube and mixed with 10-μl 2.5 M K_2_CO_3_ and then centrifuged at 13,000 rpm for 10 minutes. A total of 10-μl supernatant was collected to analyze 2,3-BPG concentration using a kit (Roche, Cat#10148334001). For erythrocyte 2,3-BPG concentration analysis, *n* = 5 mice per group were used.

### Erythrocyte BPGM activity

Blood collected from mice was centrifuged at 3,000 rpm for 5 minutes. Erythrocytes were separated from plasma as collecting at the bottom of the tube. After purification by 70% Percoll (Sigma), erythrocytes were aliquoted 60 μl per tube and frozen in liquid nitrogen, then stored in −80°C. Stored erythrocytes were lysed in customized lysis buffer (pH7.4 Tris-HCl buffer containing 0.5% Triton X-100, 1% protease inhibitor). A total of 25 μg of erythrocytes lysate was quantified by protein assay kit (Bio-Rad) and mixed with 100-μl reaction buffer (100 mM Triethanolamine pH7.6, 1 mM MgSO_4_, 4 mM ATP, 3 mM 3-phosphoglycerate, and 10-unit phosphoglycerate kinase) for 30 minutes at RT. Then, the reaction was stopped by adding 5-μl 11.63 M PCA and incubated on ice for 10 minutes. After centrifugation at 14,000 rpm at 4°C for 5 minutes, 80-μl supernatant were transferred to a new tube and subsequently mixed with 10-μl 2.5 M K_2_CO_3_ on ice for 10 minutes. A total of 10-μl supernatant were collected for analyzing BPGM activity by measuring the production of 2,3-BPG using commercial kit (Roche, Cat#10148334001). For erythrocyte BPGM activity analysis, *n* = 5 mice per group were used.

### Erythrocyte AMPK activity

Stored erythrocytes were lysed in commercial lysis buffer available in the PathScan Phospho-AMPKα (Thr172) ELISA kit (Cell Signaling Technology). RBCs lysate was quantified by protein assay kit (Bio-Rad) and then used to test the AMPK activity following the protocol in the ELISA kit. For erythrocyte AMPK activity analysis, *n* = 5 mice per group were used.

### Metabolomic profiling

Erythrocytes were stored at −80°C before metabolomic analysis. A total of 100-μl erythrocytes were extracted in a 1:10 ratio with ice-cold lysis buffer (methanol, acetonitrile, and water, 5:3:2 *v/v*). Extractions were agitated at 4°C for 30 minutes and then insoluble materials were discarded by centrifugation at 10,000 g for 15 minutes at 4°C. Moreover, 10-μl supernatant from extraction were analyzed by Ultimate 3000 UHPLC system (Thermo Fisher Scientific) coupled online with a Q Exactive system (Thermo Fisher Scientific), scanning in Full MS mode at a resolution of 70,000, scan range 65 to 900m/z, maximum injection time 200 ms, microscan 2, automatic gain control (AGC) 3 × 10^6^ ions, electrospray source voltage 4.0 kV, capillary temperature 320°C, and sheath gas 45, auxiliary gas 15, and sweep gas 0 (all nitrogen). Samples were randomized and run in positive and negative ion modes (separate runs) and separated on a Kinetex C18 column (150 × 2.1 mm, 1.7μm, Phenomenex, Torrance, CA, USA) using a 3-minute isocratic gradient at 250 μl/min (mobile phase: 5% acetonitrile, 95% 18mΩ H_2_O, 0.1% formic acid). Metabolites were converted from raw data files into mzXML format through RawConverter (Scripps Research Institute) and analyzed using Maven (Princeton University). Relative quantification of metabolites was based on integrated peak areas of extracted ion chromatograms at the MS1 level. Stability and quality control were applied by replicate injection of a technical mixture every 10 runs. For metabolomic profiling, *n* = 5 mice per group were used.

### Quantification and statistical analysis

All data are shown as mean ± SEM. Data were imported into GraphPad Prism 8 to analyze for statistical significance. Unpaired *t* test or paired *t* test according to sample distribution were used for comparing 2 groups differences. One-way ANOVA was applied to analyze mean differences among multiple groups, followed by Tukey multiple comparison test. Two-way ANOVA was used to compare data collected at from different time points, frequency, and intensity, followed by Tukey post hoc test. For all analyses, *P* < 0.05 was judged as statistically significant differences.

## Supporting information

S1 Fig Basal information of e*Adora2b*^−/−^ and control mice under different conditions. **(A)** Relative *Adora2b* mRNA levels in brain and cochlea of e*Adora2b*^−/−^, control, and WT mice. Data are expressed as mean ± SEM. *n* = 3 mice/group. **(B)** Basal hearing results from ABR tests of e*Adora2b*^−/−^ and control mice before treatment. Data are expressed as mean ± SEM. *n* = 5 mice/group. **(C–L)** Weight, ratio of spleen divided by BW, and complete blood cell results are shown. Data are expressed as mean ± SEM. *n* = 7 mice/group. **(M–O)** No statistical differences were observed between females and males in 2 genotypes and under different conditions. *n* = 3–4 mice/gender. (A, C–L) were measured by 1-way ANOVA test. (B, M–O) were tested by unpaired *t* test. No significant difference was observed. For all graphs, numerical data underlying plots are provided in S1 Data. ABR, auditory brainstem response; ADORA2B, adenosine A2B receptor; WT, wild-type.
(TIF)

S2 Fig Erythrocyte-specific deletion of ADORA2B did not affect neurons and astrocytes response in 6-month-old mice. **(A, B)** Representative images of neurons by NeuN staining in CTX (A) and HIP (B) are shown. **(C)** Representative images of astrocytes by GFAP staining in CTX and HIP are shown. **(D–G)** Quantification of neuron cell amount in CTX (D) and HIP(E) were determined by the proportion of positive NeuN area in CTX or HIP. Astrocyte amount were quantified by percentage of positive GFAP area in CTX(F) and HIP(G). Data are expressed as mean ± SEM. *n* = 5 mice/group. **(H)** Representative images of caspase-3 staining, an antigen marker of apoptotic cells, in 4 groups with little positive signal. (D-G) were assessed by 1-way ANOVA test. No significant difference was observed. (H) Representative images of caspase-3 staining, a marker of apoptotic cells, in 4 groups with little positive signal. Positive control were cochlea section from *Ada*^−/−^ mice. (A–C, H) Scale bar, 100 μm. For all graphs, numerical data underlying plots are provided in S1 Data. ADORA2B, adenosine A2B receptor; CTX, cerebral cortex; HIP, hippocampus.
(TIF)

S3 Fig Erythrocyte-specific deletion of ADORA2B did not affect neurons and astrocytes response to hypoxia. **(A, B)** Representative images of neurons visualized by NeuN staining in CTX (A) and HIP (B) are shown. Scale bar, 100 μm. **(C)** Representative images of astrocytes by GFAP staining in CTX and HIP are shown. Scale bar, 100 μm. **(D)** Representative images of caspase-3 staining, a marker of apoptotic cells, in 4 groups with little positive signal. Scale bar, 100 μm. **(E, F)** Quantification of neurons in CTX (E) and HIP (F) are represented by the proportion of NeuN positive area in CTX or HIP. Data are expressed as mean ± SEM. *n* = 5 mice/group. **(G, H)** Astrocyte amounts were quantified by percentage of positive GFAP area in CTX (G) and HIP (H). Data are expressed as mean ± SEM. *n* = 5 mice/group. (E–H) were analyzed by unpaired *t* test. No significant difference was observed. For all graphs, numerical data underlying plots are provided in S1 Data. ADORA2B, adenosine A2B receptor; CTX, cerebral cortex; HIP, hippocampus.
(TIF)

S4 Fig Mouse erythrocyte metabolic screening heatmap. Heatmap showing relative abundance of metabolites in major metabolism pathways in the erythrocytes of control or e*Adora2b*^−/−^ mice with normoxia or hypoxia treatment. *n* = 5 mice/group. Numerical data underlying heatmap are provided in S1 Data. ADORA2B, adenosine A2B receptor.
(TIF)

S1 Table Metabolites identified from erythrocyte through metabolic screening. List of 222 metabolites has been identified through metabolic screening in the erythrocytes of *eAdora2b*^−/−^ and control mice with or without hypoxia treatment. Median values of each metabolite were calculated from each group. *n* = 5 for each group. Numerical data underlying table are provided in S1 Data. ADORA2B, adenosine A2B receptor.
(XLSX)

S1 Data. Numerical data used in figure preparation (XLSX)

## Abbreviations

2,3-BPG: 2,3-bisphosphoglycerate
ABR: auditory brainstem response
AD: Alzheimer disease
ADORA1: adenosine A1 receptor
ADORA2A: adenosine A2A receptor
ADORA2B: adenosine A2B receptor
ADORA3: adenosine A3 receptor
AGC: automatic gain control
AICAR: 5-aminoimidazole-4-carboxamide ribonucleotide
ARD: age-related disease
AMPK: AMP-activated protein kinase
AN: auditory nerve
BM: Barnes Maze test
BMC: bone marrow cell
BPGM: bisphosphoglycerate mutase
BW: body weight
CNS: central nervous system
CTX: cerebral cortex
EpoR: erythropoietin receptor
HBOT: hyperbaric oxygen treatment
HGB: hemoglobin
HIP: hippocampus
HL: hearing loss
IF: immunofluorescence
IL: interleukin
iNOS: inducible nitric oxide synthase
MCH: mean corpuscular hemoglobin
MCV: mean corpuscular volume
NOR: novel object recognition
O_2_: oxygen
PCA: principal component analysis
qRT-PCR: quantitative PCR
RBC: red blood cell
RT: room temperature
SGN: spiral ganglion neuron
SPL/BW: spleen weight over body weight ratio
TDT: Tucker-Davis Technologies
TNF-α: tumor necrosis factor alpha
WBC: white blood cell
WT: wild-type
