# Supplementary figures and images for: Erythrocyte adenosine A2B receptor prevents cognitive and auditory dysfunction by promoting hypoxic and metabolic reprogramming
Source: PLoS Biol. 2021 Jun 17;19(6):e3001239. doi: 10.1371/journal.pbio.3001239 (PMC8211187; doi:10.1371/journal.pbio.3001239)

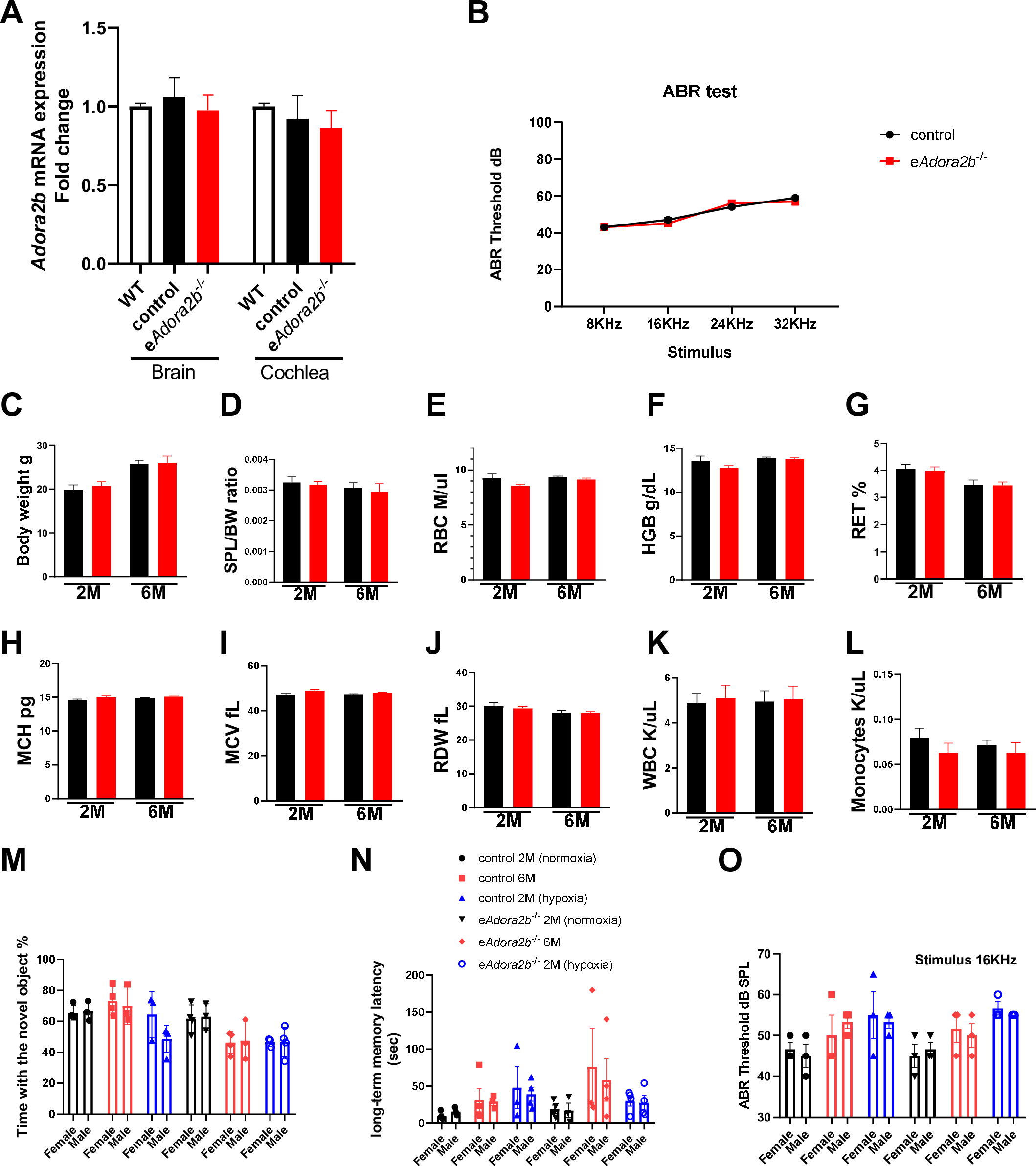

Supplement: S1 Fig — (A) Relative Adora2b mRNA levels in brain and cochlea of eAdora2b−/−, control, and WT mice. Data are expressed as mean ± SEM. n = 3 mice/group. (B) Basal hearing results from ABR tests of eAdora2b−/− and control mice before treatment. Data are expressed as mean ± SEM. n = 5 mice/group. (C–L) Weight, ratio of spleen divided by BW, and complete blood cell results are shown. Data are expressed as mean ± SEM. n = 7 mice/group. (M–O) No statistical differences were observed between females and males in 2 genotypes and under different conditions. n = 3–4 mice/gender. (A, C–L) were measured by 1-way ANOVA test. (B, M–O) were tested by unpaired t test. No significant difference was observed. For all graphs, numerical data underlying plots are provided in S1 Data. ABR, auditory brainstem response; ADORA2B, adenosine A2B receptor; WT, wild-type. (TIF) [file pbio.3001239.s001.tif]

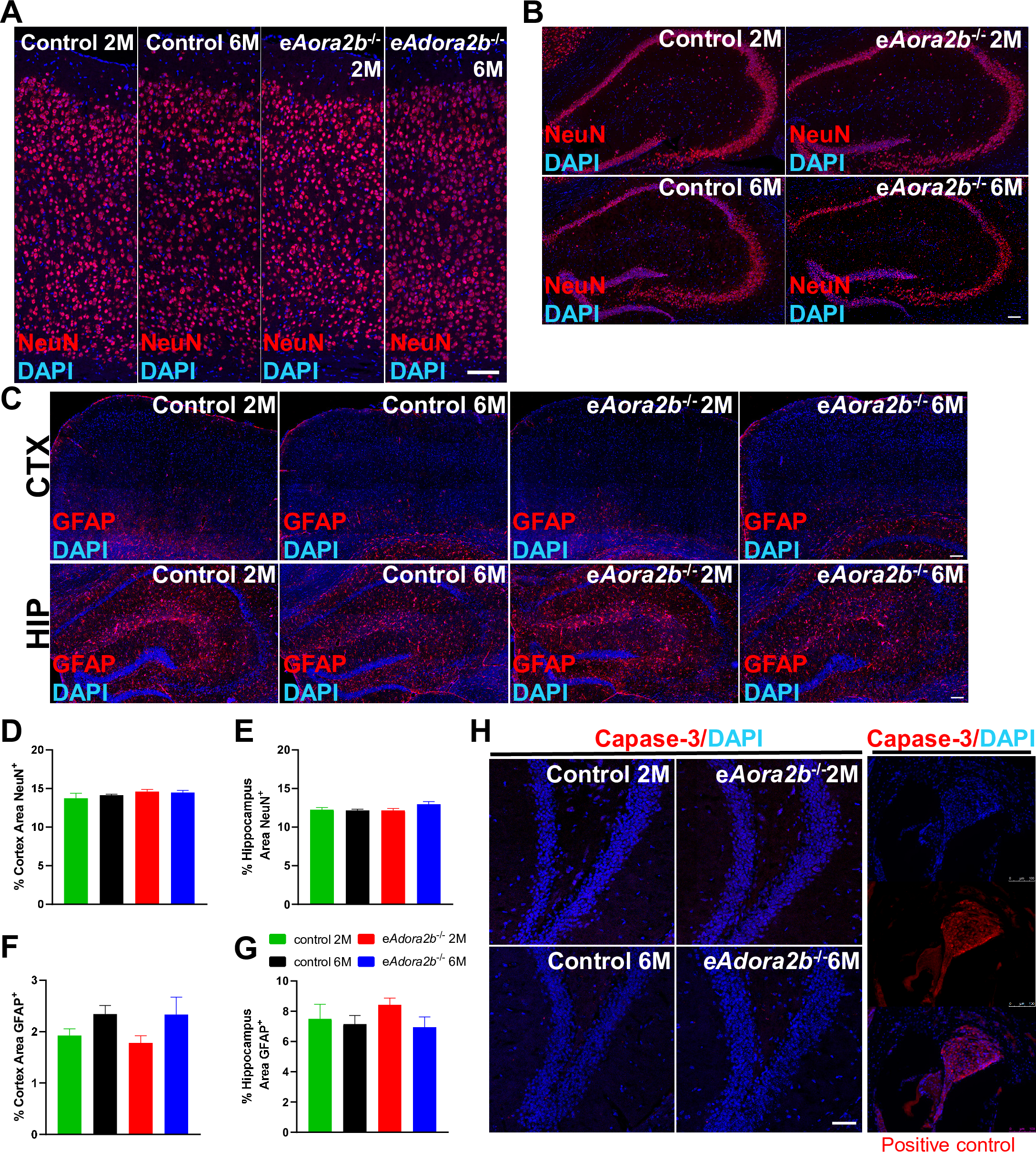

Supplement: S2 Fig — (A, B) Representative images of neurons by NeuN staining in CTX (A) and HIP (B) are shown. (C) Representative images of astrocytes by GFAP staining in CTX and HIP are shown. (D–G) Quantification of neuron cell amount in CTX (D) and HIP(E) were determined by the proportion of positive NeuN area in CTX or HIP. Astrocyte amount were quantified by percentage of positive GFAP area in CTX(F) and HIP(G). Data are expressed as mean ± SEM. n = 5 mice/group. (H) Representative images of caspase-3 staining, an antigen marker of apoptotic cells, in 4 groups with little positive signal. (D-G) were assessed by 1-way ANOVA test. No significant difference was observed. (H) Representative images of caspase-3 staining, a marker of apoptotic cells, in 4 groups with little positive signal. Positive control were cochlea section from Ada−/− mice. (A–C, H) Scale bar, 100 μm. For all graphs, numerical data underlying plots are provided in S1 Data. ADORA2B, adenosine A2B receptor; CTX, cerebral cortex; HIP, hippocampus. (TIF) [file pbio.3001239.s002.tif]

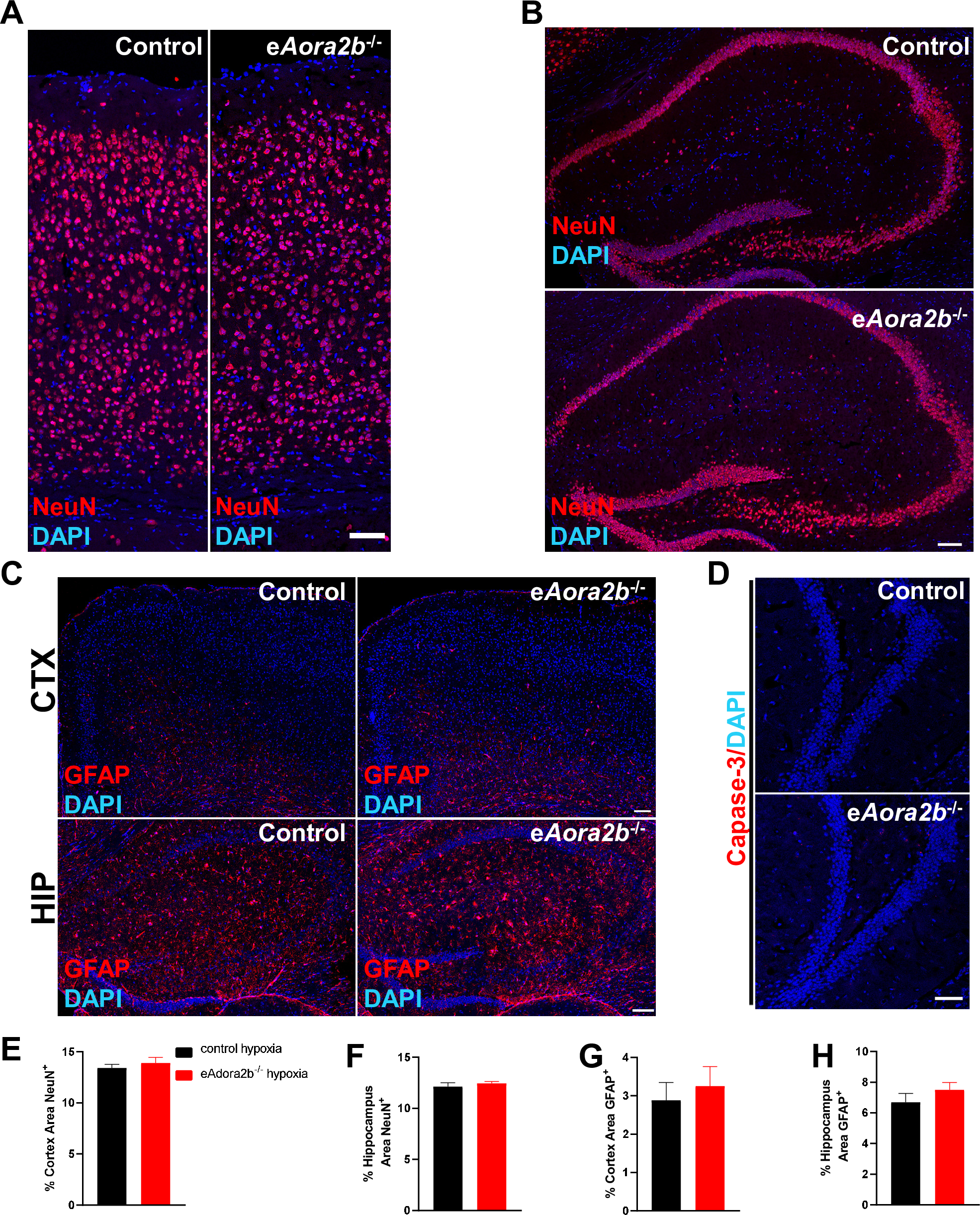

Supplement: S3 Fig — (A, B) Representative images of neurons visualized by NeuN staining in CTX (A) and HIP (B) are shown. Scale bar, 100 μm. (C) Representative images of astrocytes by GFAP staining in CTX and HIP are shown. Scale bar, 100 μm. (D) Representative images of caspase-3 staining, a marker of apoptotic cells, in 4 groups with little positive signal. Scale bar, 100 μm. (E, F) Quantification of neurons in CTX (E) and HIP (F) are represented by the proportion of NeuN positive area in CTX or HIP. Data are expressed as mean ± SEM. n = 5 mice/group. (G, H) Astrocyte amounts were quantified by percentage of positive GFAP area in CTX (G) and HIP (H). Data are expressed as mean ± SEM. n = 5 mice/group. (E–H) were analyzed by unpaired t test. No significant difference was observed. For all graphs, numerical data underlying plots are provided in S1 Data. ADORA2B, adenosine A2B receptor; CTX, cerebral cortex; HIP, hippocampus. (TIF) [file pbio.3001239.s003.tif]

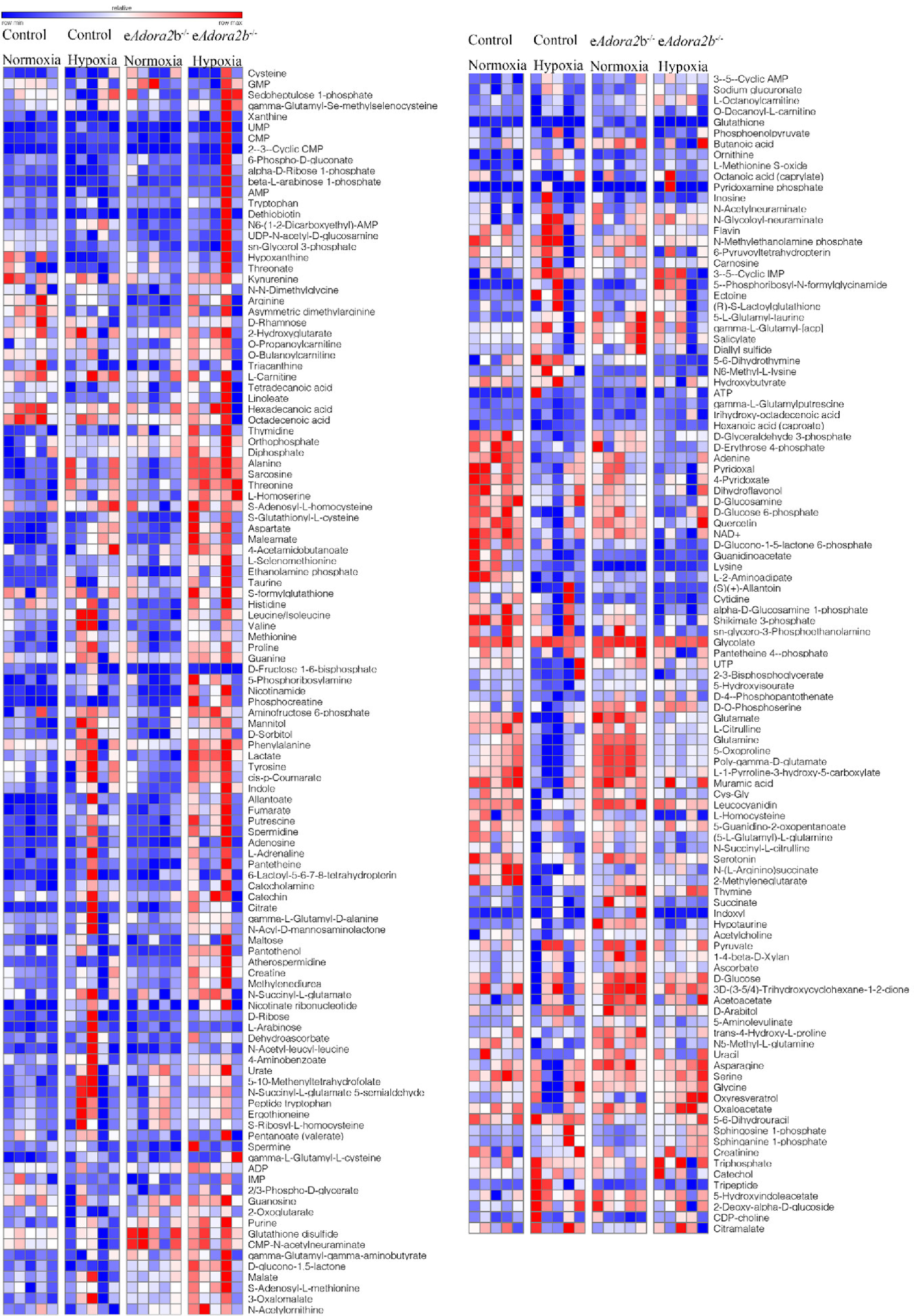

Supplement: S4 Fig — Heatmap showing relative abundance of metabolites in major metabolism pathways in the erythrocytes of control or eAdora2b−/− mice with normoxia or hypoxia treatment. n = 5 mice/group. Numerical data underlying heatmap are provided in S1 Data. ADORA2B, adenosine A2B receptor. (TIF) [file pbio.3001239.s004.tif]
